# Evolutionary and demographic consequences of temperature-induced masculinization under climate warming: the effects of mate choice

**DOI:** 10.1186/s12862-021-01747-3

**Published:** 2021-02-04

**Authors:** Edina Nemesházi, Szilvia Kövér, Veronika Bókony

**Affiliations:** 1 Lendület Evolutionary Ecology Research Group, Plant Protection Institute, Centre for Agricultural Research, Eötvös Loránd Research Network, Herman Ottó út 15, 1022 Budapest, Hungary; 2 Conservation Genetics Research Group, Department of Ecology, University of Veterinary Medicine Budapest, István utca 2, 1078 Budapest, Hungary; 3 Konrad Lorenz Institute of Ethology, Department of Interdisciplinary Life Sciences, University of Veterinary Medicine, Savoyenstr. 1a, 1160 Vienna, Austria

**Keywords:** Sexual selection, Sex-ratio selection, Climate change, Mate choice, Sex reversal

## Abstract

**Background:**

One of the dangers of global climate change to wildlife is distorting sex ratios by temperature-induced sex reversals in populations where sex determination is not exclusively genetic, potentially leading to population collapse and/or sex-determination system transformation. Here we introduce a new concept on how these outcomes may be altered by mate choice if sex-chromosome-linked phenotypic traits allow females to choose between normal and sex-reversed (genetically female) males.

**Results:**

We developed a theoretical model to investigate if an already existing autosomal allele encoding preference for sex-reversed males would spread and affect demographic and evolutionary processes under climate warming. We found that preference for sex-reversed males (1) more likely spread in ZW/ZZ than in XX/XY sex-determination systems, (2) in populations starting with ZW/ZZ system, it significantly hastened the transitions between different sex-determination systems and maintained more balanced adult sex ratio for longer compared to populations where all females preferred normal males; and (3) in ZW/ZZ systems with low but non-zero viability of WW individuals, a widespread preference for sex-reversed males saved the populations from early extinction.

**Conclusions:**

Our results suggest that climate change may affect the evolution of mate choice, which in turn may influence the evolution of sex-determination systems, sex ratios, and thereby adaptive potential and population persistence. These findings show that preferences for sex-linked traits have special implications in species with sex reversal, highlighting the need for empirical research on the role of sex reversal in mate choice.

## Background

As the Earth's climate is warming, the persistence of wildlife populations is threatened by climate-driven changes in abiotic and biotic factors [1], among which the sex ratio can play a crucial role. In some species, offspring sex is determined by environmental temperatures experienced during a sensitive period of gonadal development (environmental or temperature-dependent sex determination). In such species, climate warming may increasingly distort the populations' sex ratios, leading to loss of genetic diversity and adaptive potential via reduced effective population sizes and, ultimately, to demographic collapse [2]. However, species that have genetic sex determination, where the sex chromosomes or other genetic elements trigger male or female sexual development, are not safe from climate-induced sex-ratio shifts either. Sex reversals, where genetically female individuals become phenotypic males or vice versa, have been observed in various ectothermic taxa including fish [3], amphibians [4–7], reptiles [8], and invertebrates [9]. With increasing interest in this topic, sex reversal has been demonstrated in a growing number of species, suggesting that this phenomenon may be widespread [8]. In many of these cases, sex reversal is caused by extremely low or high environmental temperatures during early individual development. Exposure to unusually high temperature causes masculinization in many fish and anurans, and feminization in some urodelans and lizards [3, 4, 8, 10]. In natural populations of such species, global climate change may potentially distort the sex ratios and, with significant temperature rise, may lead to population extinction [11]. Furthermore, theoretical models suggest that climate change may cause, via temperature-induced sex reversals, drastic changes in the population's genetic constitution, including novel genotypes, sex-chromosome extinction, and turnovers of the sex-determination system e.g. from genetic to temperature-dependent sex determination as well as from female-heterogametic (ZW/ZZ system; ZW females, ZZ males) to male-heterogametic (XX/XY system; XX females, XY males) sex-chromosome systems [11–15].

**Fig. 1:**
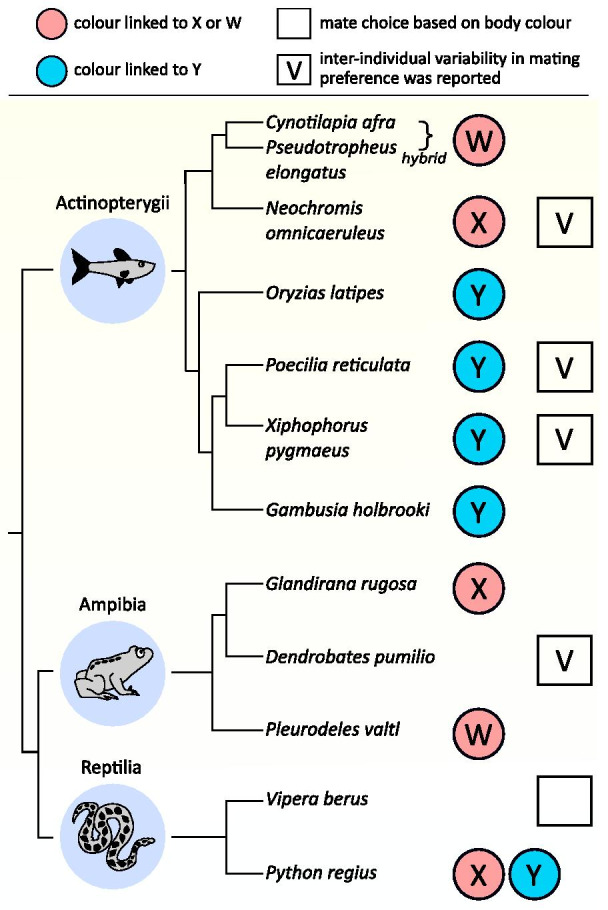
Empirical examples on sex-linkage of, and mating preference for, body colour in taxa liable to temperature-induced sex reversal. For more examples and for references, see Additional file 1: Table S1

These theoretical models of climate-driven sex reversals assumed that sex-reversed individuals are not recognized during mating, their reproductive success depending only on their fertility. However, because sex-reversed individuals can differ from normal individuals in fecundity and offspring sex ratio [16–18], individuals may benefit from taking sex reversal into account during mate choice. Choosy females then could influence the genetic sex ratio of their offspring: while mating with a normal male would result in 50% male (XY or ZZ) and 50% female (XX or ZW) offspring, a sex-reversed (XX or ZW) male would produce 100% (XX) or 75% (ZW and WW) female offspring. Such mate-choice decisions might adjust offspring sex ratios to the distorted population sex ratio and, ultimately, might save the population from extinction. Hence, preference for sex-reversed males may potentially alter the outcomes of climate change. To our knowledge, this idea has never been addressed by theoretical studies, despite the possibility that sex-reversed individuals may be recognized by conspecifics and distinguished from normal individuals during mate choice [19, 20]. Because sex reversal is challenging to study empirically, and only in recent years has it started to draw attention from field biologists and behavioural ecologists, no empirical study has yet tested the role of temperature-induced sex-reversal in mating success. However, increasing evidence shows that sex-reversed and normal individuals differ in morphology, physiology, and behaviour [7, 21–23], all of which may affect mate choice. Furthermore, the sex-chromosome genotype of sex-reversed individuals differs from that of normal individuals of the same phenotypic sex, so they can be distinguished on the basis of phenotypic traits linked to sex chromosomes. For example, there are several taxa with sex-chromosome-linked (hereafter referred to as sex-linked) body colour genes (Fig. 1, Additional file 1: Table S1) [24–26]. In such species, females may recognize sex-reversed (genetically female) males by the absence of a Y-linked male colour trait (in XX/XY system) or by the presence of a W-linked female colour trait (in ZW/ZZ system). Many other sexually selected traits, such as pheromones, body size, and song are also often sex-linked (Additional file 1: Table S1) [27, 28]. All this suggests that the implications of sex reversal for mate choice by sex-linked traits need to be addressed (Additional files 2, 3, 4 and 5).

**Fig. 2:**
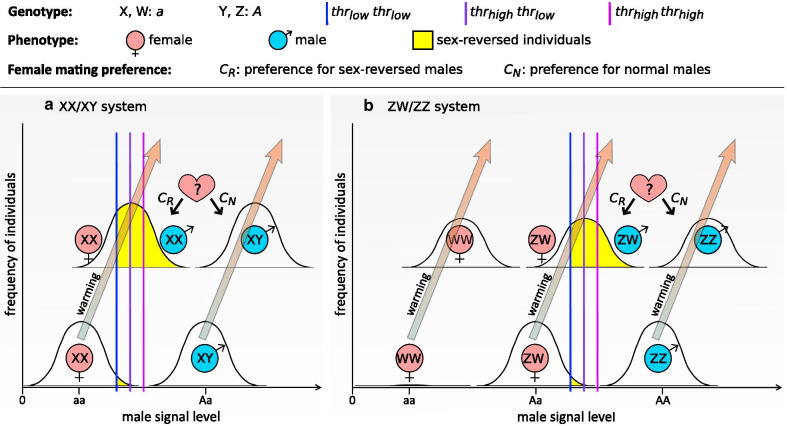
Sex as a threshold trait and manifestation of female choosiness. Sex chromosomes *A* and *a* produce different amounts of the ‘male signal’ factor. An individual becomes male if its male signal level exceeds its individual threshold defined by the individual’s *thr* genotype (according to one of the three vertical lines). Production of male signal is increased by environmental temperature experienced during a sensitive period of early individual development; the Gaussian curves represent the distribution of individual signal levels due to variation in environmental temperatures. Before climate warming, most individuals develop sexual phenotype corresponding to their sex-chromosome genotype (bottom curves in each panel), and only occasional events of masculinization occur in some *thr*_*low*_*thr*_*low*_ individuals (yellow area). Climate warming (illustrated by grey-orange arrows) increases the production of male signal by both sex chromosomes, resulting in more frequent masculinization (top curves in each panel). When both normal and sex-reversed males are present, females can choose based on their preference-allele expression (*C*_*R*_ or *C*_*N*_)

In this study we developed an individual-based theoretical model to investigate the role of female choice in the evolutionary and demographic consequences of temperature-induced sex reversal in a warming climate (Fig. 2). We focused on masculinization because this seems to be the more frequent response to high developmental temperatures across ectothermic vertebrates [3, 4, 8, 10]. We assumed that females can distinguish between normal and sex-reversed males by sex-linked traits, and some females tend to mate with sex-reversed males due to a genetically encoded preference. We hypothesised that mating preference for sex-reversed males could be beneficial for females and, consequently, spread in the population under climate warming, because females that chose sex-reversed males would produce more daughters, relative to those choosing normal males, in the increasingly male-biased population. We tested this prediction, and we investigated how preference for sex-reversed males influences the adult sex ratio, the evolutionary changes in the sex-determination system, and the duration of population persistence. Although populations can no longer persist once climate has become so hot that all females get masculinized, we predicted that sexual selection may cause earlier extinction if it biases the adult sex ratio toward males. Alternatively, if sexual selection leads to less male-biased sex ratios, it may protect the population from demographic and environmental stochasticity and thereby from premature extinction. We investigated scenarios of long-term climate warming (which occurred several times over Earth's history [29, 30]), because models of contemporary climate change project continuous warming for the twenty-first century despite the recent hiatus [31, 32].

## Results

### Consequences of climate warming without preference for sex-reversed males

**Fig. 3:**
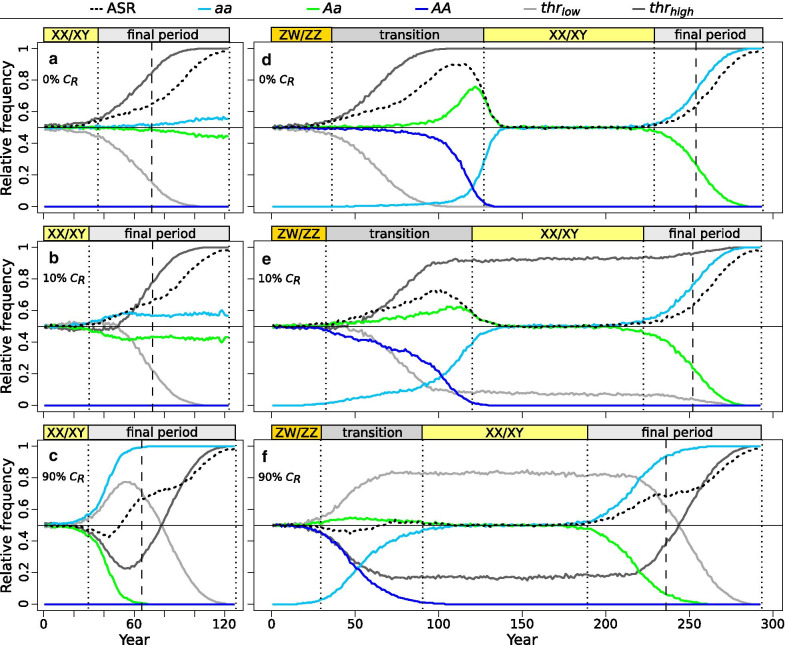
Changes in relative frequency of males (ASR), sex-chromosome genotypes and threshold alleles among adults. On the left: simulations starting with XX/XY system with scenarios 0% *C*_*R*_ (**a**), 10% *C*_*R*_ (**b**) and 90% *C*_*R*_ (**c**). On the right: simulations starting with ZW/ZZ system with scenarios 0% *C*_*R*_ (**d**), 10% *C*_*R*_ (**e**) and 90% *C*_*R*_ (**f**). Each curve indicates median values calculated from 100 runs (see Additional file 4: Fig. S2 and Fig. S5–8 for graphical results of all simulations). Vertical dotted lines indicate the end of each sex-determination period and dashed lines indicate the start of ultimate population decline. Generation time was 3 years. Year zero refers to the start of climate warming

When we assumed that all females preferred normal males (scenario 0% *C*_*R*_), a continuous rise in environmental temperatures resulted in changes in population structure in terms of genotypes and phenotypic sexes, leading to evolutionary switches between sex-determination systems and, ultimately, to population extinction (Fig. 3a,d). When starting with an XX/XY system, the increasing frequency of masculinized individuals quickly skewed the ASR towards phenotypic males and resulted in a mixed sex-determination system (hereafter referred to as ‘final period’) in which phenotypic males with both *Aa* (XY) and *aa* (XX) genotypes were present (Fig. 3a). Because individuals possessing one or two copies of the *thr*_*high*_ allele were less susceptible to temperature-induced masculinization, and thus were able to remain females during the earliest stages of climate warming, this allele spread and usually became fixed in the population (Fig. 3a, Additional file 4: Fig. S2a). However, even homozygotes for the *thr*_*high*_ allele started to masculinize as climate got hotter, leading to population extinction after ca. 42 generations (Fig. 3a).

When starting with a ZW/ZZ system, the earliest stages of climate warming caused similar increases in ASR and frequency of the *thr*_*high*_ allele as in the XX/XY system (Fig. 3d). However, the ZW/ZZ system adapted to climate change by transitioning into an XX/XY system as follows. Increasing frequency of masculinization led to a mixed sex-determination system in which phenotypic males with both *AA* (ZZ) and *Aa* (ZW, corresponding to XY) genotypes were present (Fig. 3d). During this transitional mixed sex-determination period, the ASR became strongly male-biased, which decreased the effective population size such that in some runs the population barely escaped extinction (Additional file 4: Fig. S3d). Under male-biased ASR, sex-ratio selection favoured the reproduction of non-preferred *Aa* males (masculinized individuals) because those produced less male-biased offspring for two reasons. First, mating between two *Aa* individuals produced fewer genotypic males (25% *AA* offspring, in contrast to 50% from normal matings). Second, such mating events produced 25% *aa* (WW) offspring, a novel genotype in the ZW/ZZ system which was resistant to temperature-induced masculinization as long as climate warming was mild, due to its genetically low levels of male signal. These “resistant females” rapidly accumulated because increasingly high numbers of sex-reversed males could reproduce (as the proportion of masculinized individuals increased among phenotypic males, more and more females were forced to accept them). Because all females preferred normal *AA* males, and the female phenotype became more and more restricted to the *aa* genotype, dis-assortative mating occurred between the two homozygote genotypes. This produced an excess of *Aa* genotypes, while genotype *AA* disappeared from the population because *aa* females could not produce *AA* offspring. This way, the population transitioned to an XX/XY system, where all phenotypic females had the *aa* genotype (WW became XX) and all phenotypic males had the *Aa* genotype (ZW became XY), producing 0.5 progeny sex ratios and returning the ASR to 0.5 (Fig. 3d). This system persisted until *aa* individuals started to get masculinized by the ever-increasing temperatures, once again skewing the ASR towards phenotypic males. During this ‘final period’, the frequency of mating between masculinized and normal *aa* individuals increased, thereby chromosome *A* (Y) became rare or even extinct. When no phenotypic females were left, the population died out, ca. 98 generations after the start of climate warming (Fig. 3d).

### Effects of preference for sex-reversed males

**Fig. 4:**
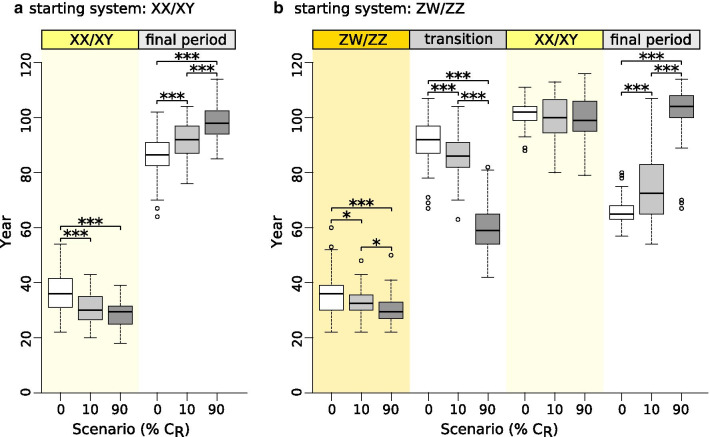
Length of consecutive sex-determination periods in each scenario starting with XX/XY system (**a**) or ZW/ZZ system (**b**). Each box plot shows the distribution (thick middle line: median, box: interquartile range; whiskers extend to the most extreme data points within 1.5 × interquartile range from the box) of the results of 100 runs. Significant pairwise differences are indicated above the box plots as: * 0.01 < p < 0.05, *** p < 0.001 (for details see Additional file 1: Table S3). Generation time was 3 years. Year zero refers to the start of climate warming

**Fig. 5:**
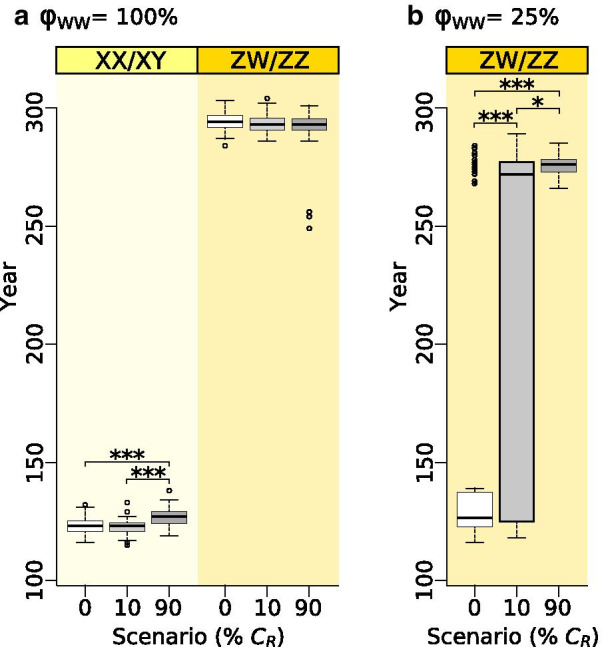
Population persistence in our main scenarios (starting with either XX/XY or ZW/ZZ system; (**a**) and in the sensitivity test with ZW/ZZ initial system in which the survival of the WW genotype (φ) was reduced to 25% compared to the survival of genotypes ZW and ZZ (**b**). Each box plot shows the distribution of the results of 100 runs (thick middle line: median, box: interquartile range; whiskers extend to the most extreme data points within 1.5 × interquartile range from the box). Significant pairwise differences are indicated above the box plots as: * 0.01 < p < 0.05, *** p < 0.001. Generation time was 3 years. Year zero refers to the start of climate warming

**Fig. 6:**
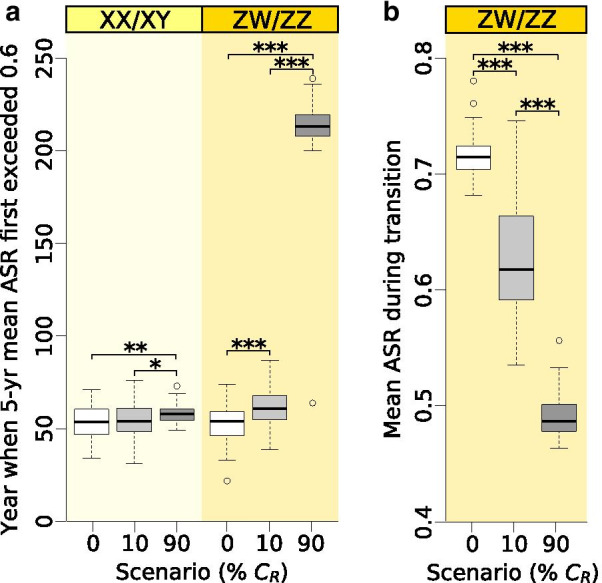
Differences in adult sex ratios (ASR, proportion of males) among scenarios. Each box plot shows the distribution (thick middle line: median, box: interquartile range; whiskers extend to the most extreme data points within 1.5 × interquartile range from the box) across 100 runs for the year when mean ASR across 5 consecutive years first exceeded 0.6, starting with either XX/XY or ZW/ZZ system (**a**), and the mean ASR during the transition period between the initial ZW/ZZ and the subsequent XX/XY system (**b**). Significant pairwise differences are indicated above the box plots as: * p < 0.05, ** p < 0.01, *** p < 0.001 (for details see SI1 Table S3). Generation time was 3 years. Year zero refers to the start of climate warming

When we allowed females to vary in mating preference, the presence of allele *C*_*R*_ significantly changed the temporal dynamics of the above processes and the magnitude of ASR skew (Figs. 3, 4 , 5 and 6, Additional file 1: Table S3). The evolutionary switches between sex-determination systems happened faster, resulting in shorter initial period in both starting systems, shorter transition period between ZW/ZZ and XX/XY, and longer final period, whereas the duration of the XX/XY system that evolved from the ZW/ZZ system was not affected (Fig. 4). These changes were greater when the initial frequency of the *C*_*R*_ allele was higher (Fig. 4). Ultimately, these changes did not alter the population’s extinction time when starting from a ZW/ZZ system (although a few populations in the 90% C_R_ scenario died out prematurely; see Additional file 4: Fig. S3f), but the XX/XY system starting with a widespread *C*_*R*_ allele survived slightly longer (Fig. 5). Notably, however, all these differences in duration were biologically small, averaging only a few generations (Additional file 1: Table S3).

Average ASR was not affected by the presence of allele *C*_*R*_ during the initial ZW/ZZ and XX/XY periods, nor during the XX/XY period evolved from the ZW/ZZ system (Additional file 1: Table S3). As these periods were defined by the scarcity of sex-reversed males and thus little variation in female choice, ASR remained near 0.5 (Fig. 3). However, during the mixed sex-determination period following the initial sex-chromosome system, the presence and frequency of allele *C*_*R*_ significantly influenced the timing and extent of ASR skew towards males (Fig. 3, Fig. 6, Additional file 1: Table S3). When the initial frequency of the *C*_*R*_ allele was 10%, it had little effect on ASR in the XX/XY system (Fig. 3b), but populations starting with ZW/ZZ system had significantly less male-biased ASR during the transitional period between ZW/ZZ and XX/XY (on average, 63% males instead of 72%) and reached 0.6 ASR about 3 generations later than populations where all females preferred normal males (Fig. 3e, Fig. 6). When the initial frequency of allele *C*_*R*_ was 90%, it had even greater effects on ASR (Fig. 3f, Fig. 6). First, in both systems, ASR decreased slightly below 0.5 temporarily after the initial sex-determination system ended, returning then to 0.5 (Fig. 3c, f). After that, ASR remained close to 0.5 throughout the transition period following the ZW/ZZ system, keeping the population in a balanced sex ratio for ca. 50 generations longer compared to the other two scenarios (Fig. 3d-f, Fig. 6a). Starting with the XX/XY system, ASR increased to 0.6 slightly later when allele *C*_*R*_ was widespread in the initial population compared to scenario 0% *C*_*R*_ (Fig. 3a-c, Fig. 6a), although the slope of ASR increase was alternatingly steeper and shallower before shifting ultimately to 1 (Fig. 3a-c).

### Background of preference effects: sex-ratio selection and sexual selection

**Fig. 7:**
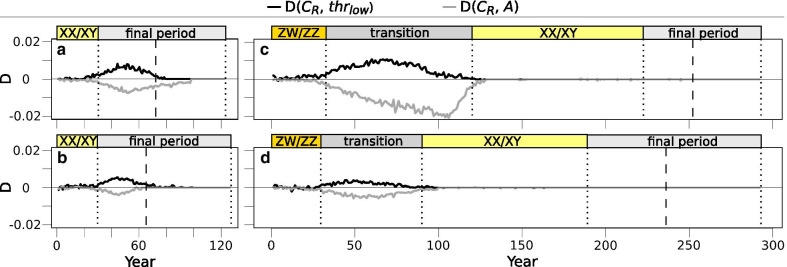
Linkage disequilibrium coefficient (D) between preference allele *C*_*R*_ and either the threshold allele *thr*_*low*_ or sex chromosome *A.* On the left: simulations starting with XX/XY system with scenarios 10% *C*_*R*_ (**a**) and 90% *C*_*R*_ (**b**). On the right: simulations starting with ZW/ZZ system with scenarios 10% *C*_*R*_ (**c**) and 90% *C*_*R*_ (**d**). Each curve indicates median values calculated from 100 runs. Vertical dotted lines indicate the end of each sex-determination period and the dashed lines indicate the start of ultimate population decline. Note that negative linkage disequilibrium (LD) with *A* is equivalent with positive LD with *a*. Year zero refers to the start of climate warming

**Fig. 8:**
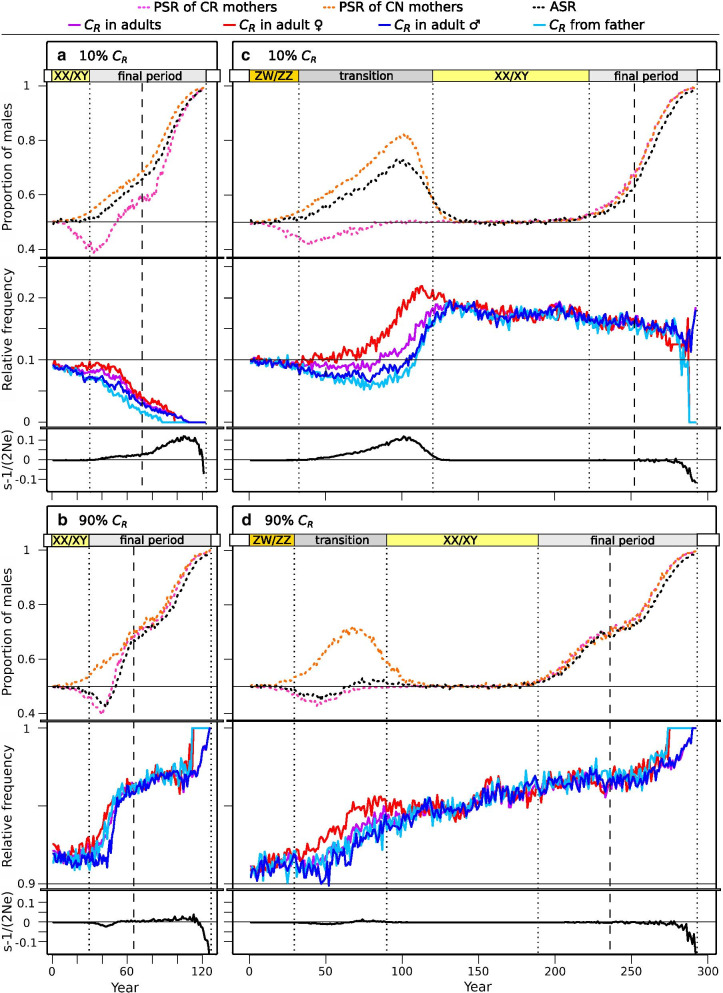
Consequences of mate choice: progeny sex ratio, inheritance of allele *C*_*R*_ and sex-ratio selection acting on allele *C*_*R*_. On the left: simulations starting with XX/XY system with scenarios 10% *C*_*R*_ (**a**) and 90% *C*_*R*_ (**b**). On the right: simulations starting with ZW/ZZ system with scenarios 10% *C*_*R*_ (**c**) and 90% *C*_*R*_ (**d**). In each panel, the top three curves show the changes in adult sex ratio (ASR) and progeny sex ratio (PSR) of mothers preferring sex-reversed and normal males (CR and CN mothers, respectively); the four curves in the middle show the relative frequency of allele *C*_*R*_ across and among sexes (*C*_*R*_ in adults, *C*_*R*_ in adult females, *C*_*R*_ in adult males) and the relative frequency of *C*_*R*_ among preference alleles inherited by offspring from their fathers (*C*_*R*_ from father; note how it differs from the *C*_*R*_ in adult males). Mothers passed on allele *C*_*R*_ with a relative frequency corresponding to its presence among adult females (not shown). The bottom curve of each panel shows the strength of sex-ratio selection (*s*) relative to the effective population size (N_e_), expressed as *s*–1/(2N_e_) (this value is shown instead of *s* itself, because the effect of genetic drift can override *s* in small populations). Each curve indicates median values calculated from 100 runs (see Additional file 4: Fig. S4 for the relative frequencies of *C*_*R*_ in all individual runs). Vertical dotted lines indicate the end of each sex-determination period and dashed lines indicate the start of ultimate population decline. Note that the top, middle, and bottom part of each panel has three different Y axes and with different scales to facilitate visibility. Year zero refers to the start of climate warming

Starting with XX/XY system and a rare (10%) dominant *C*_*R*_ allele, the accumulation of masculinized individuals was slightly sped up by the presence of females preferring them. Because individuals possessing the *thr*_*low*_ allele were the first to masculinize, they were the ones chosen by females possessing *C*_*R*_, leading to positive LD between *thr*_*low*_ and *C*_*R*_ (Fig. 7a) and keeping *thr*_*low*_ from decreasing for several decades (Fig. 3b). This resulted in earlier transition into the mixed system of the final period. However, the rare *C*_*R*_ allele had little if any effect on ASR, because its spread was selected against for the following reasons. During the first decades, masculinized individuals (*aa* males) and the females choosing them (females with *C*_*R*_) produced female-biased offspring (all *aa* genotypes, facing relatively low masculinization rates at this early stage), which was not beneficial because the ASR was still close to 0.5 (Fig. 8a). As ASR became more and more male-biased, sex-ratio selection increasingly favoured *C*_*R*_ (Fig. 8a), but sexual selection acted against it for the following reasons. Because females carrying *C*_*R*_ preferred to mate with sex-reversed males (*aa*, i.e. those without chromosome *A*), negative LD arose between *C*_*R*_ and *A* (Fig. 7a)*.* As the majority of females did not possess allele *C*_*R*_ and thus preferred normal males, sexual selection favoured males with chromosome *A*, and thereby acted against *C*_*R*_ due to the negative LD between *C*_*R*_ and *A.* Thus, males carrying *C*_*R*_ were less likely to become fathers than those without *C*_*R*_ (i.e. proportion of *C*_*R*_ was lower among the alleles passed on by fathers than among the alleles in adult males; see light and dark blue lines in Fig. 8a). Therefore, the frequency of allele *C*_*R*_ decreased significantly (on average by 0.032, 95% confidence interval [CI]: 0.025–0.039, t_99_ = 8.56, p < 0.001) from the start of the final mixed-system period until the beginning of ultimate population decline, and the *C*_*R*_ allele generally vanished before population extinction (Fig. 3b, Additional file 4: Fig. S4a).

When the XX/XY system started with a common (90%) recessive *C*_*R*_ allele, more drastic changes occurred. Since the majority of females preferred sex-reversed males, as soon as the latter started to spread they flooded the population with female-biased offspring that skewed the ASR towards females (Fig. 3c) and passed on *C*_*R*_ and *thr*_*low*_. This increased the frequency of both alleles significantly (Figs. 3c,  8b) and created LD between C_R_ and *A* and between C_R_ and *thr*_*low*_ (Fig. 7b). Due to the widespread preference for *aa* males and the positive LD between *a* and *C*_*R*_, the relative frequency of *C*_*R*_ was higher in males becoming fathers than in the adult male population (Fig. 8b). Therefore, sexual selection spread *C*_*R*_ (Fig. 8b; average increment in *C*_*R*_ frequency was 0.029, 95% CI: 0.020–0.039, t_99_ = 6.132, p < 0.001) despite the disadvantage of *C*_*R*_ in sex-ratio selection while climate warming was relatively mild. The spread of *C*_*R*_ was accompanied by a rapid shift towards male-biased ASR because the widepsread preference for sex-reversed males increased the frequency of *thr*_*low*_ to ca. 80% (Fig. 3c). Notably, sexual selection for the lack of chromosome *A* (Y) eradicated it from the population about 20 generations after the start of climate warming, leaving only *aa* genotypes (Fig. 3c) and ending all selection on *C*_*R*_ (Fig. 8b). At this point, the role of the sex-chromosome-linked sex-determining locus was taken over by the autosomal threshold locus, as individuals possessing (more copies of) *thr*_*high*_ could resist masculinization for some time. During this time, sex-ratio selection favoured *thr*_*high*_ and started to spread it until fixation, which temporarily slowed the increase of masculinization rate and ASR (Additional file 4: Fig. S5c, Fig. 3c). This resulted in maintaining somewhat higher effective population size compared to scenarios with no or rare *C*_*R*_ (Additional file 4: Fig. S3 and Fig. S9), and thereby slightly increased the population's survival time (Fig. 7a).

Starting with ZW/ZZ system and dominant allele *C*_*R*_ at 10% frequency, the simulations resulted in similar LD of *C*_*R*_ with chromosome *a* (W) and allele *thr*_*low*_ (Fig. 7c) as described above. This slowed the decrease of *thr*_*low*_ and accelerated the accumulation of *aa* genotypes and thereby the transition into XX/XY system (Fig. 3e). Because not all females preferred normal (*AA*) males, this scenario produced a milder excess of *Aa* genotypes during the transition period (Fig. 3e) compared to the scenario without *C*_*R*_ (Fig. 3d). The slightly better mating success of masculinized individuals resulted in faster accumulation of "resistant females" (*aa*), slowing the ASR increase and keeping it less male-biased during the transition period (Fig. 3e, Fig. 6b). Nevertheless, ASR was skewed enough so sex-ratio selection favoured parents that produced less male-biased offspring, i.e. females choosing masculinized individuals, thus the frequency of *C*_*R*_ almost doubled by the end of the transition period (Fig. 8c; during ZW/ZZ to XX/XY transition, relative frequency of allele *C*_*R*_ increased on average by 0.076, 95% CI: 0.059–0.093; paired t-test: t_99_ = 9.0, p < 0.001). Once the *AA* genotype went extinct, both sex-ratio selection for allele *C*_*R*_ (and chromosome *a*; Fig. 7c) and sexual selection against it ended, and the further processes followed a similar course as in the scenario without *C*_*R*_ (Fig. 3d-e).

When the ZW/ZZ system started with the recessive *C*_*R*_ allele at 90% frequency, the effects seen in the previous scenario became stronger, via similar mechanisms. Since the majority of females preferred masculinized individuals, the accumulation of *aa* genotypes and the transition to XX/XY system were even faster (Fig. 3f, Fig. 4b). During the transition period, there was only a small excess of *Aa* genotypes (Fig. 3f) because *AA* males were soon replaced by *Aa* males as the latter spread and were preferred by most females (who increasingly had the *aa* genotype). The frequency of *thr*_*low*_ increased greatly due to the widespread preference for individuals with low masculinization thresholds (Fig. 3f). Since masculinized individuals enjoyed high mating success, ASR became slightly female-biased at the start of the transition period, but soon returned to 0.5 and remained close to it afterwards (Figs. 3f,  6b) because the production of "resistant females" compensated for the increasing masculinization rate. The frequency of allele *C*_*R*_ slightly increased during the transition period (by 0.021 on average, 95% CI: 0.012–0.030, t_99_ = 4.707, p < 0.001; Fig. 8d) due to two reasons: 1) sexual selection (i.e. the more preferred sex-reversed males carried allele *C*_*R*_ and passed it on to the next generation with higher than expected probability: Fig. 8d), and 2) LD with chromosome *a* (W) and allele *thr*_*low*_ (Fig. 7d). Overall preference for sex-reversed males kept the frequency of *thr*_*low*_ around 80% until nearly 75 generations after the start of climate warming, causing a relatively early and sudden ASR increment after the XX/XY system ceased to persist (Fig. 3f). Thereafter, sex-ratio selection favoured allele *thr*_*high*_ over *thr*_*low*_, but could not prevent a relatively early population decline compared to the other scenarios (Fig. 3f, Additional file 4: Fig. S3f). Thus, when chromosome *A* went extinct and the role of the sex-determining locus was taken over by the autosomal threshold locus, *thr*_*high*_ was already close to fixation and so this new sex-determination system persisted for only a few generations before the population died out (Fig. 3f).

### Sensitivity analysis

The above-described effects of *C*_*R*_ were very similar in the following cases: when we assumed 1) a multiallelic *thr* locus (instead of biallelic) or 2) intermediate inheritance of *C* alleles (instead of fully dominant/recessive), or 3) when the value of *C*_*N*_ was set to 0.5, encoding indiscriminate mating, or 4) when viability of WW offspring was reduced by 25% or 50% (Additional file 5: Fig. S10–S15). Changes in relative frequency of *C*_*R*_ were also very similar in these cases (Additional file 5**:** Fig. S10–S15), except that it showed little if any change over time when initial frequency of *C*_*R*_ was 10% and *C*_*N*_ encoded indiscriminate mating (Additional file 5: Fig. S11, S12).

However, when WW viability was reduced by 75%, presence or absence of *C*_*R*_ made a significant difference (Additional file 5**:** Fig. S16; Fig. 5). When *C*_*R*_ was absent, the population could transition to an XX/XY system and persist for ca. 270 years in only 26% of runs; the rest died out after ca. 120 years (Fig. 5). When *C*_*R*_ was present, it saved the population from early extinction by enabling the switch to XX/XY system in 56% of runs when *C*_*R*_ was rare and in 100% of runs when *C*_*R*_ was widespread (Fig. 5). Due to high mortality of WW offspring, relative frequency of *C*_*R*_ started to decrease in both scenarios, but it increased rapidly just before the end of transition when ASR became highly male-biased (Additional file 5**:** Fig. S16).

In contrast, when we assumed that the WW genotype was not viable, the ZW/ZZ system always behaved like the XX/XY system (no transition in sex determination; population extinction after ca. 120 years of warming) and the presence of *C*_*R*_ had very little effect on the shifting of ASR towards males (Additional file 5: Fig. S17). Relative frequency of both the rare and the widespread *C*_*R*_ decreased steadily in these scenarios, as females mating with sex-reversed males lost 25% of their offspring because of being WW (Additional file 5: Fig. S17).

## Discussion

We investigated how female preference for sex-reversed males would affect evolution in an increasingly masculinizing environment. Our simulations showed that the presence and frequency of such a preference allele (*C*_*R*_) influenced both the temporal dynamics of evolution of sex-determination systems and the changes in adult sex ratio across a wide range of circumstances, and under certain conditions it also affected the timing of population extinction. Furthermore, we found that a rare, dominant *C*_*R*_ allele may spread in populations with ZW/ZZ sex-determination sytem more likely than in populations with XX/XY system. We discuss each of these main findings in detail below.

In our simulations, increasing masculinization rate under continuous climate warming resulted in a process where different sex-determination systems replaced one another. This agrees with the findings of previous models [11–14] and experimental data [16] suggesting that climate change may cause turnovers between different sex-determination systems and thus may have contributed to the variability of sex-determination systems across ectothermic vertebrates [33, 53, 54]. Our present results demonstrate for the first time that the speed of these turnovers may be enhanced by sexual selection if females can recognize and prefer to mate with sex-reversed males. Specifically, when the *C*_*R*_ allele was present in our simulations, the initial sex-determination system (either XX/XY or ZW/ZZ) evolved sooner into a mixed sex-determination system, and when the initial system was ZW/ZZ, the transitional mixed system also evolved sooner into an XX/XY system. These effects were stronger when the frequency of *C*_*R*_ was higher. Furthermore, a widespread *C*_*R*_ allele facilitated a turnover in the XX/XY system that was not seen when *C*_*R*_ was rare or absent: the original male-determining sex chromosome went extinct and its role was taken over by an autosome (harbouring the original threshold locus). These results suggest the idea that, over the evolutionary past with alternatingly warmer and colder climates, variation in female preferences for sex-linked traits might have catalized the diversification of sex-determination systems in taxa that are prone for sex reversals. Further models could test this idea by simulating climate warming followed by a period of stable climate [15].

Because WW individuals could resist masculinization longer, production of viable WW offspring was key to the transiton from ZW/ZZ to XX/XY system in our model. Therefore, reduced WW viability had major impacts on the outcomes in our sensitivity tests. Complete WW lethality erased any difference in ASR and persistence between ZW/ZZ and XX/XY systems and any effect of *C*_*R*_. By contrast, 25% WW viability resulted in strikingly divergent fates with and without *C*_*R*_, whereby majority of the populations possessing *C*_*R*_ persisted for more than twice as long as populations without *C*_*R*_. This shows that sexual selection for sex-reversed males may prevent premature extinction in certain circumstances. However, when the WW genotype was at least fairly viable (50% or higher viability), *C*_*R*_ had very little effect on population persistence in our simulations. The latest possible extinction time was determined by the values of allele *thr*_*high*_ and *b*_*sig*_ (which were fixed in our simulations): when climate warming caused masculinization in all *thr*_*high*_ homozygotes, no more female offspring could be produced, after which the population could persist only as long as the remaining females survived. Populations with ZW/ZZ initial system persisted longer when they could transition into an XX/XY system, but within simulations of each initial system, females disappeared from the populations at roughly the same time in all scenarios, regardless of the frequency of the *C*_*R*_ preference allele. The only exception was that extinction happened a few years later when *C*_*R*_ had high frequency in populations with XX/XY initial system. In this scenario, after chromosome Y disappeared, only individuals with low endogenous male signal levels (i.e. XX) remained, and therefore there were slightly more females (i.e. non-masculinized XX individuals) in the final years before population extinction. Although population persistence may be prolonged by occurrence of new, mutant alleles causing lower male signal levels or higher threshold levels, we did not allow for such mutations in our model because we assumed that the rapid climate change would not provide enough time for new mutants to appear and spread before population extinction, for the following reasons. First, the realistically small population size in our model would restrict the number of new mutants to less than one (assuming a mutation rate of 10^–5^ over 100 generations in a population of 200 adults). Second, rare new mutant alleles would have a high chance of random loss, and even if they spread they would be selected for only in periods with unbalanced ASR, whereas they would face counter-selection in periods with balanced ASR. In accordance with this, the time needed for evolutionary changes of sex-determination systems was typically long (hundreds or thousands of generations) in previous models that allowed for new mutations [12–14]. Bearing this caveat in mind, our results suggest that female preference for sex-reversed males alone cannot grant much longer persistence for a population under continuous, rapid climate warming if the WW genotype does not suffer from markedly increased mortality rate. However, in our simulations, population persistence was not threatened by anything other than climate-driven masculinization. In reality, further effects of climate change or other perturbations may also harm the populations [55], and resilience against these perturbations might be affected by *C*_*R*_, as discussed next.

We found that female preferences can have a significant effect on changes of adult sex ratio during climate warming. While the male to female ratio was around 1:1 during ZW/ZZ and XX/XY sex-determination periods, it markedly shifted towards males in the mixed periods in scenarios where all females favoured normal males. By contrast, presence of allele *C*_*R*_ prolonged the time before the ASR became strongly male-biased (except when starting from an XX/XY system with a rare *C*_*R*_). Furthermore during the transition from ZW/ZZ to XX/XY system, *C*_*R*_ kept the ASR less male-biased (when *C*_*R*_ was rare or when WW had reduced viability) or close to a healthy 0.5 (when *C*_*R*_ was widespread). This way, the presence of allele *C*_*R*_ helped maintain a higher effective population size when populations without *C*_*R*_ suffered population bottlenecks. Populations with less biased ASR and higher effective population size are more likely to survive environmental perturbations such as anthropogenic habitat loss and disease outbreaks that currently parallel and interact with the effects of global climate change [55]. In this respect, our results suggest that ZW/ZZ populations with widespread preference for sex-reversed males might have the highest adaptive potential and the best chances to cope with contemporary climate change. Understanding the factors underlying the variance in adaptability is important because a recent meta-analysis suggests that animals' adaptive responses to climate change may often be insufficient [1].

Our present results support the previous findings that XX/XY and ZW/ZZ systems may differ in their responses to climate change [11, 14]. Which of the two systems is more resilient depends on several conditions, such as the fertility of sex-reversed males and the viability of WW individuals [11]. Here we found that the ZW/ZZ system may maintain healthier sex ratios for longer, and persist more than two times longer, than the XX/XY system if the sex-reversed males can reproduce like normal males and produce viable, fertile WW offspring that are resistant to masculinization. These conditions stand in various taxa [10, 17, 33, 41–43, 56], although our empirical knowledge on WW or masculinized individuals in nature is scarce. Moreover, most ectothermic vertebrates possess homomorphic sex chromosomes that show no signs of degeneration, suggesting that sex-reversed and WW individuals should be viable and fertile in the majority of these taxa [4, 44, 53]. Under these conditions, our results confirm that female-heterogametic systems may be less vulnerable in a masculinizing environment compared to male-heterogametic systems [11]. Our present model contributes to this picture with the novel finding that the frequency of female preference for sex-reversed males is a further condition that may lead to different effects of climate change in the two sex-chromosome systems. For example, after ca. 60 years of warming, while both systems had similarly male-biased ASR when *C*_*R*_ was rare, the ZW/ZZ system had much more balanced ASR than the XX/XY system when *C*_*R*_ was widespread (Fig. 3). Furthermore, our results show that the two systems respond to climate warming identically when the WW genotype is lethal, and also when WW viability is poor and *C*_*R*_ is absent, but the ZW/ZZ system is more likely to outlive the XX/XY system even with poorly viable WW if *C*_*R*_ is present.

A further difference between the two sex-chromosome systems in our model was seen in the changes in *C*_*R*_ frequency when *C*_*R*_ was competing with an allele encoding preference for normal males. We found that a rare *C*_*R*_ allele spread in the population when the initial sex-determination system was ZW/ZZ, but tended to disappear instead when sex determination was initially XX/XY. The major difference between the two systems was that sex-ratio selection that favoured allele *C*_*R*_ was stronger in the ZW/ZZ system, because females carrying *C*_*R*_ produced WW offspring that were resistant to masculinization, thus their progeny sex ratios were more advantageous when ASR was male-biased. This stronger sex-ratio selection in the ZW/ZZ system counteracted the prevailing sexual selection that acted against *C*_*R*_ in both systems due to the widespread preference for normal males and to the LD between *C*_*R*_ and the sex chromosomes. Thus, our results suggest that a rare autosomal preference for sex-reversed males is more likely to spread in ZW/ZZ than XX/XY systems. This finding parallels previous theoretical studies that showed that the ZW/ZZ system is particularly prone to evolve sex-linked preferences for sexually antagonistic traits [46] and a new male ornament is more protected against random loss in ZW/ZZ compared to XX/XY systems [47].

By contrast, when *C*_*R*_ was widespread in the population, sexual selection in both systems favoured sex-reversed males and consequently allele *C*_*R*_ that was more frequent in such males. In these scenarios, sex-ratio selection played virtually no role in the spread of *C*_*R*_, because adult sex ratio hardly deviated from 0.5 as long as both normal and sex-reversed males were present. Both sexual selection and sex-ratio selection have long been known to be major driving forces of evolution [49, 57, 58]; and these forces together can even lead to speciation [59]. Occurrence of novel combinations of genetic sex and sex-linked coloration under sexual selection can lead to rapid sympatric speciation [60]; thus, our findings raise the possibility that climate-driven sex reversals might contribute to speciation. Because sex-ratio selection, which spread *C*_*R*_ in our simulated populations, was due to the masculinizing effect of rising environmental temperature, our results demonstrate that climate change may influence the evolution of female mate choice. This finding parallels the conclusions of a modelling study showing that environmental pollution may disrupt sexual selection and thereby decrease population fitness [61]. Taken together, these theoretical results highlight that various forms of ongoing anthropogenic environmental change worldwide may be driving changes in mating preferences, which then can have knock-on consequences on adaptive potential and population viability.

## Conclusions

Our main conclusion is that sexual selection for sex-linked traits may influence the effects of climate change on the demography and evolution of populations with temperature-sensitive sex development. This provokes several further questions for future studies. On the one hand, the genetic architecture of sexually selected traits and the genetic and environmental determinants of sex are poorly known for many taxa. To assess how much wildlife is at risk by climate change, we need more empirical information filling these knowledge gaps. Our study highlights that mate choice for sex-linked traits may have crucial consequences in species with sex reversal, so we urgently need empirical research to test if conspecifics recognize sex-reversed individuals and behave differently towards them. On the other hand, we also need theoretical studies on how further factors affect our projections. For example, in species where the temperature reaction norm is non-linear, climate warming may lead to ZZ feminization and ultimately to the loss of the W chromosome [15]. In bearded dragons (*Pogona vitticeps*), for instance, ZZ individuals develop into females at high temperatures, and surprisingly, these sex-reversed females enjoy a fecundity advantage over normal females [16]. In such case, males might prefer sex-reversed females, which may complicate the effects of climate warming similarly to what we found here for female choice. Spatially heterogeneous temperatures may further complicate these outcomes, either by high dispersal dampening the sex-ratio bias at the metapopulation level, or by low dispersal leading to restricted habitat use and reduced population growth rate [62]. As growing evidence suggests that sex reversal is more likely widespread in ectotherms rather than a rare oddity [8], exploring the complexity of its consequences is an important emerging research avenue.

## Methods

### The model

We followed the approach of recent theoretical models on the evolution of sex determination, where sex has been assumed to be a threshold trait: a phenotypically discrete trait (i.e. male or female) determined by individual threshold sensitivity for the endogenous level of a non-discrete factor referred to as ‘male signal’ [12–14]. In our model, individuals are diploid and carry a pair of sex chromosomes and two autosomal loci; all three loci are inherited independently of each other (Additional file 1: Table S2). Sex chromosomes are denoted by *A* and *a*, corresponding to Z and W in a ZW/ZZ system and to Y and X in an XX/XY system, respectively. Each sex chromosome harbours a sex-determinant locus that encodes male signal: *A* causes production of the male signal at 1.5 times higher level (*sig*_*A*_) than *a* does (*sig*_*a*_) under the same environmental conditions (for graphical explanation, see Fig. 2). Male signal expression increases with environmental temperature, such that the individual level of male signal expression (*sig*_*indiv*_) in an *Aa* individual is calculated with Eq. 1:

1 $${sig}_{indiv}= {sig}_{A}+{sig}_{a}+ {sig}_{env}$$

where *sig*_*env*_ is the exogenous level of male signal due to environmental temperatures (see below). All genotypes have the same temperature sensitivity in male signal production (i.e. paralell reaction norms). We followed Grossen and colleagues [13] in assuming that male signal increases monotonically with temperature, based on the empirical observations of masculinising effects of high temperatures and feminising effects of low temperatures in several amphibians and fish [4, 33], although we note that counter-examples exist and non-linear temperature reaction norms are also possible [14, 15].

The autosomal locus *thr* encodes the individual threshold for *sig*_*indiv*_ that needs to be exceeded in order to develop male reproductive organs (otherwise, the individual becomes female). We calculated the individual's threshold value as the sum of the values of the two alleles that the individual carried at the *thr* locus. Although sex determination in temperature-sensitive systems is often assumed to have a polygenic basis [34, 35], there is very little empirical evidence either *pro* or *contra* [36–38]; and the findings of the seminal models of temperature-sensitive sex determination were not sensitive to the assumption of one versus more loci [34]. Therefore, for simplicity we assume that only two *thr* alleles at a single locus are present in the population (however, we explored additional simulations with 10 *thr* alleles as sensitivity tests). Allele values on locus *thr* are set according to the sex-determination system operating in the initial population (see Fig. 2 and Additional file 1: Table S2). Threshold value of homozygotes for the *thr*_*low*_ allele is above the average male signal level of the normal female genotype (*Aa* in system ZW/ZZ and *aa* in system XX/XY) but just below its maximum signal level realized at the temperature range before climate warming (Fig. 2) [14]. Value of the *thr*_*high*_ allele is set so that threshold in *thr*_*high*_ homozygotes equals the average of male signal levels that are determined by the normal female and male genotypes (*Aa* and *AA*, or *aa* and *Aa*, respectively, in ZW/ZZ and XX/XY system) under the temperature variation before climate warming (Fig. 2). All simulations start with both *thr* alleles present in the population at 0.5 frequency.

We ran simulations where the initial sex-determination system was either XX/XY or ZW/ZZ. We assumed that sex-reversed males (XX, ZW or WW) are as viable and fecund as normal males, following previous models and empirical data [33, 39, 40]. Note that our previous model predicted that 25% decrease in reproductive success of masculinized individuals had little effect on adult sex ratios and sex chromosome frequencies, whereas their sterility lead to the ZW system behaving exactly like the XY system [11]. Further, we assumed that the WW genotype (*aa* in ZW/ZZ system) is phenotypically equivalent to normal females (i.e. has the same viability, fecundity, and ability to masculinize). Empirical data support that WW individuals can be viable [10, 17, 33, 41, 42], able to reproduce [10, 42, 43] and can also be able to develop into functional males [39, 41]. Viability and fertility of sex-reversed males and WW individuals is likely in ectothermic vertebrates, because in these taxa the sex chromosomes are usually homomorphic [44]. However, in some species the WW genotype is lethal [42], so we explored the effects of reduced WW viability in additional simulations. We did not allow new mutations to occur on the *thr* locus or the sex-determinant locus, because appearance of a new mutant allele would be unlikely in our simulations due to the realistically small population size (starting with 200 adults) and relatively short evolutionary time (according to rapid contemporary climate change). Note, however, that evolution is still possible due to standing variation in temperature sensitivity [15], which was allowed in our simulations by changes in relative frequencies of the *thr* alleles.

In our model, climate is warming linearly over time, each year increasing the average temperature that the population is exposed to during the breeding season. In each year, each individual may experience a different environmental temperature during the sensitive period of its ontogeny due to spatiotemporal variation in microclimatic conditions. This variation in temperature between and within years is incorporated into our model by a set of parameters defining the exogenous levels of male signal (Additional file 1: Table S2), such that in year *t* the *sig*_*env*_ level for each individual is calculated as described in Eq. 2:

2 $${sig}_{env}\left(t\right)={b}_{sig}+{\upvarepsilon }_{B}+{\upvarepsilon }_{W}$$

where *b*_*sig*_ is the slope of the yearly increase in mean *sig*_*env*_ levels in the population, ε_*B*_ is the normally distributed error of the yearly mean *sig*_*env*_ levels (between-year climatic variance), and ε_*W*_ is the normally distributed error of individual developmental temperatures (within-year climatic variance; see Fig. 2). We did not vary the values of *b*_*sig*_, ε_*B*_, and ε_*W*_ in our simulations, but fixed each at a likely value based on empirical data. We set *b*_*sig*_ = 0.003 (0.3%) assuming no masculinization before 1970 (the start of contemporary, human-induced climate warming) and an increase to 9% masculinization by 2000, based on the first report of sex reversal in a natural amphibian population [5]. We set ε_*B*_ = 0.01 based on the standard deviation of temperature anomalies observed in the Northern Hemisphere between 1970 and 2000 [45]. We set ε_*W*_ = 0.05, a value five times higher than ε_*B*_, which is realistic based on empirical data for reptiles [15]. These settings ensure that, before the start of climate warming, a stable genetic sex-determination system (either XX/XY or ZW/ZZ) persists, with only occasional events of sex reversal in individuals experiencing unusually high temperature (resulting in ca. 0.2% masculinization rate before the start of climate warming; see Fig. 2 for visual representation). We allowed this stable state to persist for 50 years (i.e. burn-in period, with *b*_*sig*_ = 0), after which we simulated climate warming by increasing the mean value of *sig*_*env*_ each year by *b*_*sig*_. We chose this relatively rapid, linear increase in masculinization rate to model the climate warming observed in the recent past and expected in the near future [45]. Note that our model does not include temperature per se, only its effects on male signal production.

Besides the sex-determinant male signal locus, each sex chromosome harbours another locus that encodes a sexually dimorphic phenotypic trait, which for simplicity we will refer to as colour and model it as a binary trait. We assume that females can recognize normal males by a "male colour" expressed in the presence of chromosome Y (*A*) when we start with an XX/XY sex determination system, and by the absence of a "female colour" encoded on chromosome W (*a*) when the initial system is ZW/ZZ. The autosomal locus *C* determines preference in females for mating partners based on sex-linked colour, and is not expressed in males. We used an autosomal *C*, because empirical data suggest that female mating preference is more often autosomal than sex-linked (Supplementary Information in [46]), and sex-linkage would raise a complex problem because the outcome can depend on the sex chromosome to which the gene is linked [46, 47]. Values of *C* alleles determine the probability that a female would choose a normal male if both normal and sex-reversed males were equally available. We allowed a maximum of two *C* alleles in the population: allele *C*_*N*_ encoding a strong but non-exclusive preference (0.9) for normal males, and allele *C*_*R*_ encoding the same extent of preference for sex-reversed males (i.e. 0.1 probability of choosing a normal male; see Additional file 1: Table S2). In our simulations, inheritance of *C* is fully dominant/recessive, where heterozygotes show the same choosiness as homozygotes for the dominant allele do.

Starting with either XX/XY or ZW/ZZ sex-determination system, we investigated three different sexual-selection scenarios. In scenario 0% *C*_*R*_, no preference allele for sex-reversed males exists (all females are *C*_*N*_*C*_*N*_ homozygotes). In scenario 10% *C*_*R*_, the *C*_*R*_ allele is dominant and rare (relative frequency 0.1 in the starting population), thus initially only 19% of females (genotypes *C*_*R*_*C*_*R*_ and *C*_*R*_*C*_*N*_) express preference for sex-reversed males. This scenario could be realistic if, for example, males with female-like coloration occurred somewhat regularly in the near past (e.g. due to randomly occurring sex-reversals [6, 48]; see *pref_prob0* in Additional file 1: Table S2), maintaining a certain level of variance in female mating preferences. In scenario 90% *C*_*R*_, the *C*_*R*_ allele is recessive and widespread (relative frequency 0.9), thus initially 81% of females (*C*_*R*_*C*_*R*_) express preference for sex-reversed males. This latter scenario is possible if allele *C*_*R*_ has spread in the initial population by neutral processes or due to a sensory bias for the "female colour" that was generally not expressed in males while sex reversal was very rare (see *pref_prob0* in Additional file 1: Table S2) [49]. This can be seen as a similar case to the famous experiment of Basolo [50] where 93% of females of the swordless fish *Priapella olmeacae* preferred males with artificial swords. For simplicity, we only investigated the effects of relatively low and high *C*_*R*_ frequency, assuming that these two scenarios represent the range of potential effects (i.e. the effects of an initial *C*_*R*_ frequency between 10 and 90% may fall between the effects presented here). We chose to set the initially rare *C* allele to dominant because rare recessive alleles are easily lost by drift. However, we additionally examined several other scenarios, including intermediate inheritance and a *C* allele encoding lack of preference (indiscriminate mating), to assess the sensitivity of our results to these settings.

We aimed to build a relatively realistic model where a number of parameters affect the life history and demography of iteroparous animals, including the age of maturation, annual survival rates differing across life stages, fertility, environmental carrying capacity, and limited number of mating events for each individual per breeding season. We set these parameters to be representative for many amphibians using empirical data from the literature, mostly following our previous model [11], although similar parameter settings are representative for other temperature-sensitive taxa such as fish or reptiles (Additional file 1: Table S2). Every year, the population produces *N* offspring calculated using Eq. 3:

3 $$N=\mathrm{min}(Nmax;Nmother\times {fert}_{f})$$

where *Nmax* is the carrying capacity, *Nmother* is the number of adult females that found a mating partner, and *fert*_*f*_ is the average number of offspring each female can recruit in the absence of density-dependence (see Additional file 1: Table S2). For simplicity, we assume that the annual survival rate of juveniles and adults, age of maturity, and maximum life span were independent of both genotypic and phenotypic sex, except for scenarios with reduced WW viability (Additional file 1: Table S2). In our previous model on climate-driven sex reversal, the resulting changes in sex ratios and sex-chromosome genotype frequencies were not affected by changing the parameters to phenotypic-sex-dependent life history and genotypic-sex-dependent mortality [11], so we did not vary these parameters' values in the present model. Each year, adults participate in a single breeding event, during which mate choice is constrained by the availability of mating partners: within a breeding season, each female can mate with one male, while each male can mate with maximum three females ("libido"; see Additional file 1: Table S2 and Fig. S1). Females in a randomized order, one after another, choose a single mate from the pool of still available phenotypic males according to the females’ preference and relative frequency of the available male genotypes, resulting in altogether *Nmother* parent pairs. Thus, in an XX/XY system for example, a female with a dominant *C*_*R*_ allele would mate with an XY male with a probability of *C*_*R*_ × P_XY_, where *C*_*R*_ is the strength of preference for normal males and P_XY_ is the proportion of mating opportunities with XY males out of all available matings (i.e. number of available males multiplied by their remaining "libido"). Parent pair of each of the *N* offspring is chosen randomly, and each offspring randomly inherits one sex chromosome, one *thr* and one *C*_*R*_ allele from each parent, and receives a *sig*_*env*_ value, based on which its phenotypic sex is determined. For settings of all parameter values in detail and their justification see Additional file 1: Table S2. Our model was developed in R programming language, and the simulations were run in R 3.4.3 [51]. The source code is available in Additional file 2, including detailed comments in order to facilitate re-parametrization of the model as needed for future studies (for example, with our model, it is possible to explore taxon-specific scenarios, different pace of climate change and even feminization due to climate warming).

### Statistical analyses

We performed 100 runs for each scenario (specific R code for these simulations is available in Additional file 3). We compared the dynamics of sex-determination system evolution, sex ratios, and population persistence among scenarios by calculating the following values from each run. First, to identify transitions between different sex-determination systems, we assumed that in an XX/XY system (i.e. *aa* females, *Aa* males) at least 95% of the adult phenotypic males have the *Aa* genotype, while less than 5% are sex-reversed (*aa*). Similarly, a ZW/ZZ system (i.e. *Aa* females, *AA* males) persists as long as less than 5% of the adult males carry chromosome *a*. When more than 5% of phenotypic males are sex-reversed individuals, we assumed that a mixed sex-determination system is operating. We defined the endpoint of each sex-determination period as the first year after which the above conditions did not prevail anymore for 50 consecutive years (which in practice meant that those conditions never returned in that run). For example, an XX/XY period ended and a mixed system began when the proportion of sex-reversed males increased above 5% and did not drop below this value for 50 consecutive years. We chose the 50-year time window for identifying the start and end of each sex-determination period because there was a notable annual fluctuation in genotype frequencies among adult males due to the stochastic nature of our model. In practice this meant that the proportion of a given genotype would never exceed or fall below the specified value again, as masculinization rate of each genotype kept increasing in the continuously warming environment. For each simulation, we calculated the length of each sex-determination period in years, and also the year of extinction which occurred when no females were left in any age groups in the population.

We also investigated changes in adult sex ratio (ASR, the proportion of phenotypic males among adults) over evolutionary time in our simulations. We identified the sex-determination periods in which ASR deviated from 0.5 by visually inspecting our results, and for this period in each run, we calculated the average of yearly ASR values and the first year when average ASR across 5 consecutive years increased above 0.6.

For each variable above (genotype frequencies, ASR, length of each sex-determination system and population persistence), we compared the three scenarios (0% *C*_*R*_*,* 10% *C*_*R*_ and 90% *C*_*R*_) pairwise, starting the simulations either from ZW/ZZ or XX/XY system. To this end, we entered the values calculated from each run as a dependent variable in a linear model, using scenario as fixed factor, and we calculated three linear contrasts (post-hoc tests for the pairwise differences among the three scenarios) using the function ‘lsmeans’ in R package lsmeans [52]. All p-values of all these linear contrasts were adjusted simultaneously by Bonferroni correction (‘bonferroni’ method in the R function ‘p.adjust’). We chose this strict correction method to be conservative about statistical significance in our analyses. For scenarios in the sensitivity analysis with 25% WW viability, where the distribution of data did not meet the requirements of linear models, we used pairwise median tests with Bonferroni correction.

For scenarios 10% *C*_*R*_ and 90% *C*_*R*_ we evaluated if the relative frequency of allele *C*_*R*_ changed during the period when the following criteria were met: 1) co-occurrence of normal and sex-reversed males allowed females to choose between them (i.e. both normal and sex-reversed males were available with > 5% frequency among phenotypic males), and 2) the effect of drift (due to reduced effective population size, see below) on allele frequencies did not exceed the strength of sexual selection. For each run, we calculated the average relative frequency of allele *C*_*R*_ among adults across 5 years at the start and end of this period, and we compared the start and end values with a paired t-test (R function ‘t.test’) across all simulations within each scenario. The four p-values from these t-tests were adjusted simultaneously by Bonferroni correction.

To better understand the forces behind the changes of *C*_*R*_ frequency, we recorded for each year in each run the relative frequency of allele *C*_*R*_ among adult males, among adult females, and also among the offspring's preference alleles inherited from fathers and mothers separately. Also, we calculated the following values as detailed in Additional file 1. For each year in each run we calculated the effective population size (N_e_), the selection coefficient (*s*) of *C*_*R*_ resulting from sex-ratio selection, linkage disequilibrium (LD) between allele *C*_*R*_ and chromosome *A*, and LD between *C*_*R*_ and the *thr*_*low*_ allele. For each run, we recorded the year when the ultimate population decline started, i.e. the year in which N_e_ permanently decreased below the average N_e_ of the 10 years before the start of climate warming when the population was in a stable state (Additional file 1). Because our populations had overlapping generations, we estimated generation time (T; mean age of reproduction) as detailed in Additional file 1. In all scenarios, generation time was about 3 years in our simulations.

## Supplementary Information

**Additional file 1.** Additional methods and results.

**Additional file 2.** Model code.

**Additional file 3.** Simulation settings.

**Additional file 4.** Complementary figures for results.

**Additional file 5.** Sensitivity analysis.

## Data Availability

The simulated data supporting the results of this article are available in the FigShare repository, 10.6084/m9.figshare.13567322.v1. R codes of the model and the simulation settings as well as additional supporting material are included in this published article and its Additional files 1, 2, 3, 4 and 5.

## Abbreviations

*A* and * a*: Sex chromosomes. *A* is corresponding to Z or Y, while *a* is corresponding to W or X, in ZW/ZZ and XX/XY sex-determination systems, respectively
ASR: Adult sex ratio, as the proportion of phenotypic males among adults
*C*: An autosomal gene determining female mating preference. Allele *C*_*N*_ encodes preference for normal males, while allele *C*_*R*_ encodes preference for sex-reversed males
*fert*_*f*_: The average number of offspring each female can recruit in the absence of density-dependence (see Eq. 3).
LD: Linkage disequilibrium.
*N*: The number of offspring produced in the population each year (see Eq. 3)
N_e_: Effective population size
*Nmax*: Carrying capacity (Eq. 3)
*Nmother*: The number of adult females that found a mating partner (Eq. 3) P_XY_ is the proportion of mating opportunities with XY males out of all available matings
Sex-linked: Sex-chromosome-linked
*sig*: Individual level of the’male signal’ factor (*sig*_*indiv*_) depends on endogenus (*sig*_*A*_ ans *sig*_*a*_, produced by sex chromosomes *A* and *a*, respectively) and exogenous (*sig*_*env*_) components. The environment-dependent component *sig*_*env*_ is calculated from the slope of yearly increase of mean *sig*_*env*_ due to climate warming (*b*_*sig*_), the between-year climatic variance (ε_*B*_) and the within-year climatic variance (ε_*W*_). For details see Eqs. 1 and 2.
*thr*: An autosomal locus encoding individual threshold for the “male signal” factor in our model. Two alleles determining either lower or higher threshold occur on it (*thr*_*low*_ and *thr*_*high*_, respectively)
