# Supplementary material for: Evolutionary and demographic consequences of temperature-induced masculinization under climate warming: the effects of mate choice
Source: BMC Ecol Evol. 2021 Feb 4;21:16. doi: 10.1186/s12862-021-01747-3 (PMC7860201; doi:10.1186/s12862-021-01747-3)
Supplement: Supplementary file 4 — Additional file 4. Complementary figures for results. [file 12862_2021_1747_MOESM4_ESM.pdf]

Supporting Information 4 - Graphs showing 100 runs per scenario

Evolutionary and demographic consequences of temperature-induced masculinization under climate warming: the effects of mate choice.

Nemesházi E[1,2,3], Kövér Sz[2], Bókony V[1]

[1] Lendület Evolutionary Ecology Research Group, Plant Protection Institute, Centre for Agricultural Research, Eötvös Loránd Research Network, Herman Ottó út 15, 1022 Budapest, Hungary. E-mail: nemeshazi.edina@atk.hu, bokony.veronika@atk.hu

[2] Conservation Genetics Research Group, Department of Ecology, University of Veterinary Medicine Budapest, István utca 2, 1078 Budapest, Hungary

[3] Konrad Lorenz Institute of Ethology, Department of Interdisciplinary Life Sciences, University of Veterinary Medicine, Savoyenstr. 1a, A-1160 Vienna, Austria

Table of contents:

Figures showing specified results of 100 runs of each scenario:

Fig S2. Relative frequency of the thr_{low} allele

Fig S3. Effective population size

Fig S4. Relative frequency of preference allele C_R

Fig S5. Masculinization rate

Fig S6. Relative frequency of genotype *aa* among adults

Fig S7. Relative frequency of genotype *Aa* among adults

Fig S8. Adult sex ratio

Figure showing median values across 100 runs:

Fig S9. Median effective population size during the last decades before extinction in originally XX/XY populations

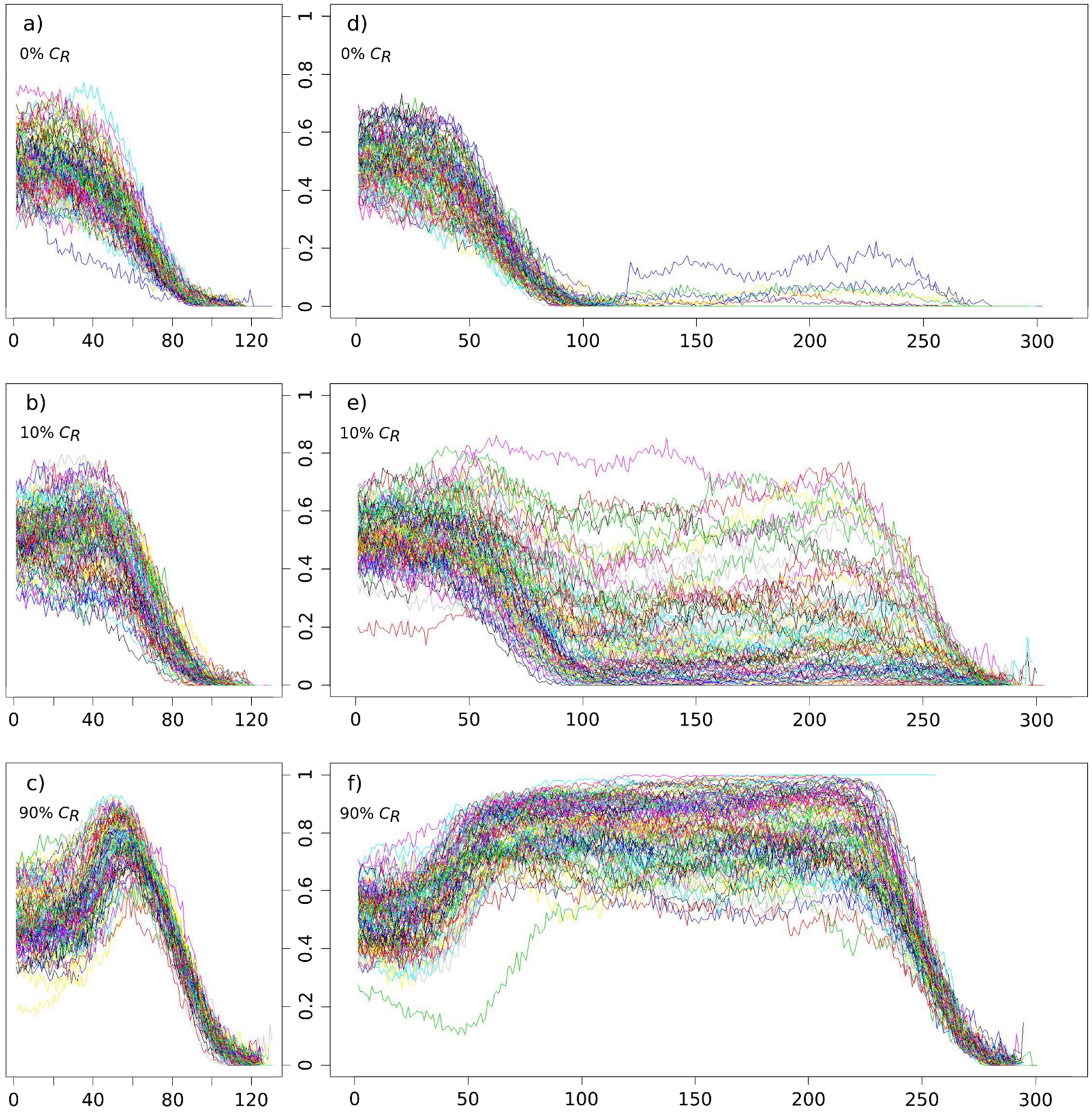

Fig S2. Relative frequency of the *thr_{low}* allele. X axis: years of climate warming. Y axis: relative frequency. On the left: scenarios 0% C_R (a), 10% C_R (b) and 90% C_R (c) starting with XX/XY system. On the right: scenarios 0% C_R (d), 10% C_R (e) and 90% C_R (f) starting with ZW/ZZ system. Curves indicate separate runs (100 repeated runs per scenario).

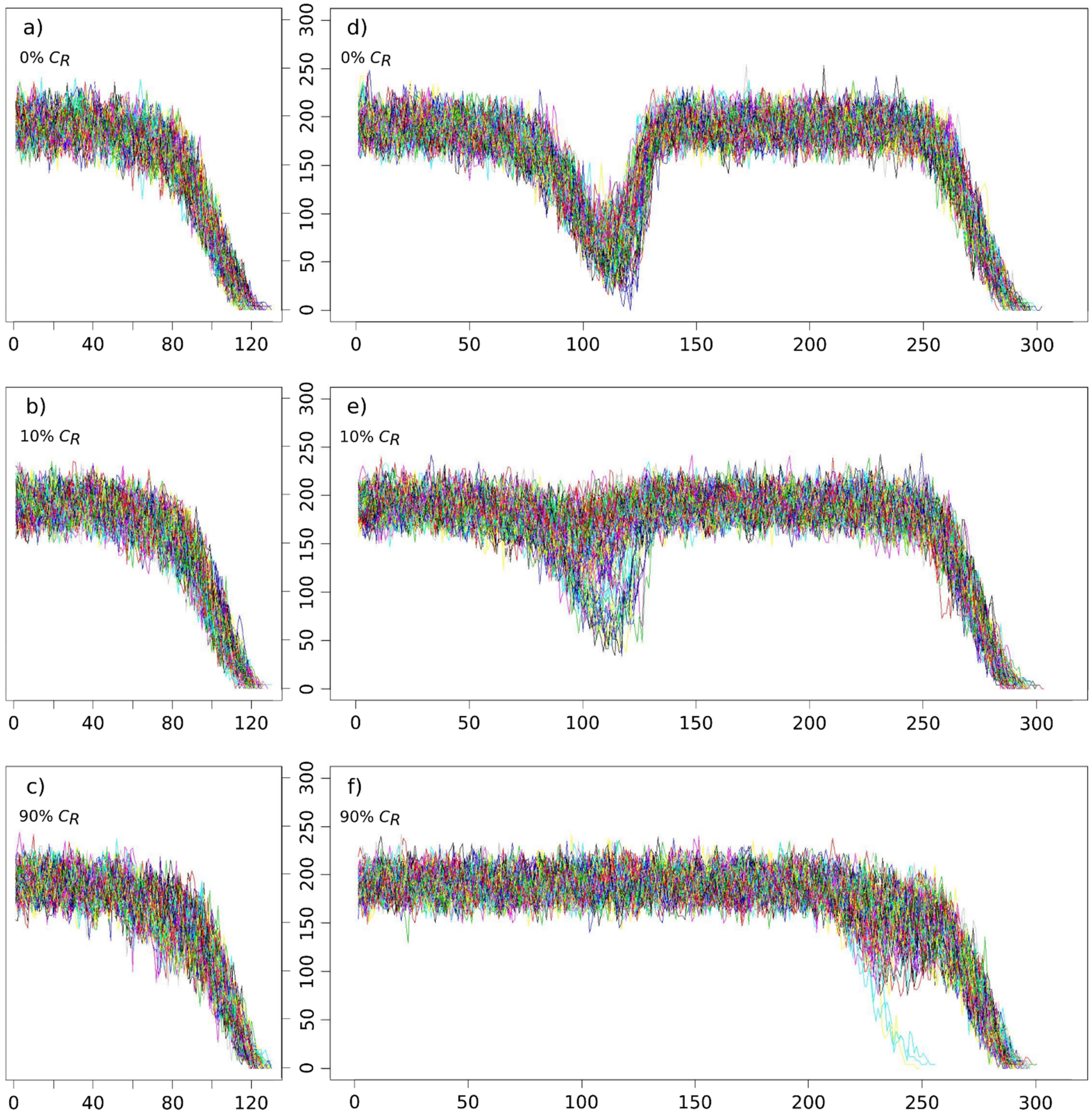

Fig S3. Effective population size. X axis: years of climate warming. Y axis: effective population size (calculated from the number of adult males and adult females present in the population). On the left: scenarios 0% C_R (a), 10% C_R (b) and 90% C_R (c) starting with XX/XY system. On the right: scenarios 0% C_R (d), 10% C_R (e) and 90% C_R (f) starting with ZZ/ZW system. Curves indicate separate runs (100 repeated runs per scenario).

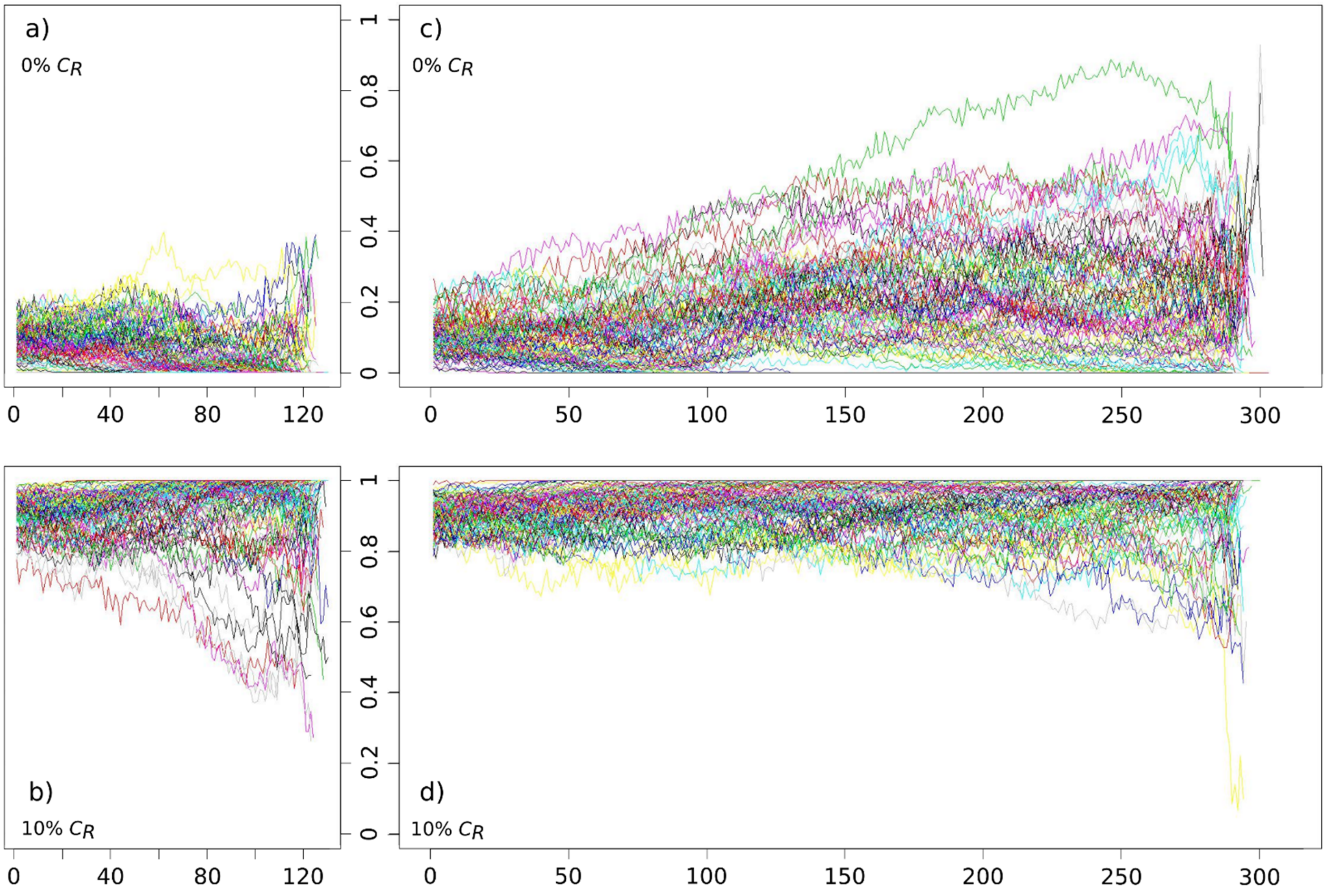

Fig S4. Relative frequency of preference allele C_R. X axis: years of climate warming. Y axis: relative frequency. On the left: scenarios 10% C_R (a) and 90% C_R (b) starting with XX/XY system. On the right: scenarios 10% C_R (c) and 90% C_R (d) starting with ZW/ZZ system. Curves indicate separate runs (100 repeated runs per scenario).

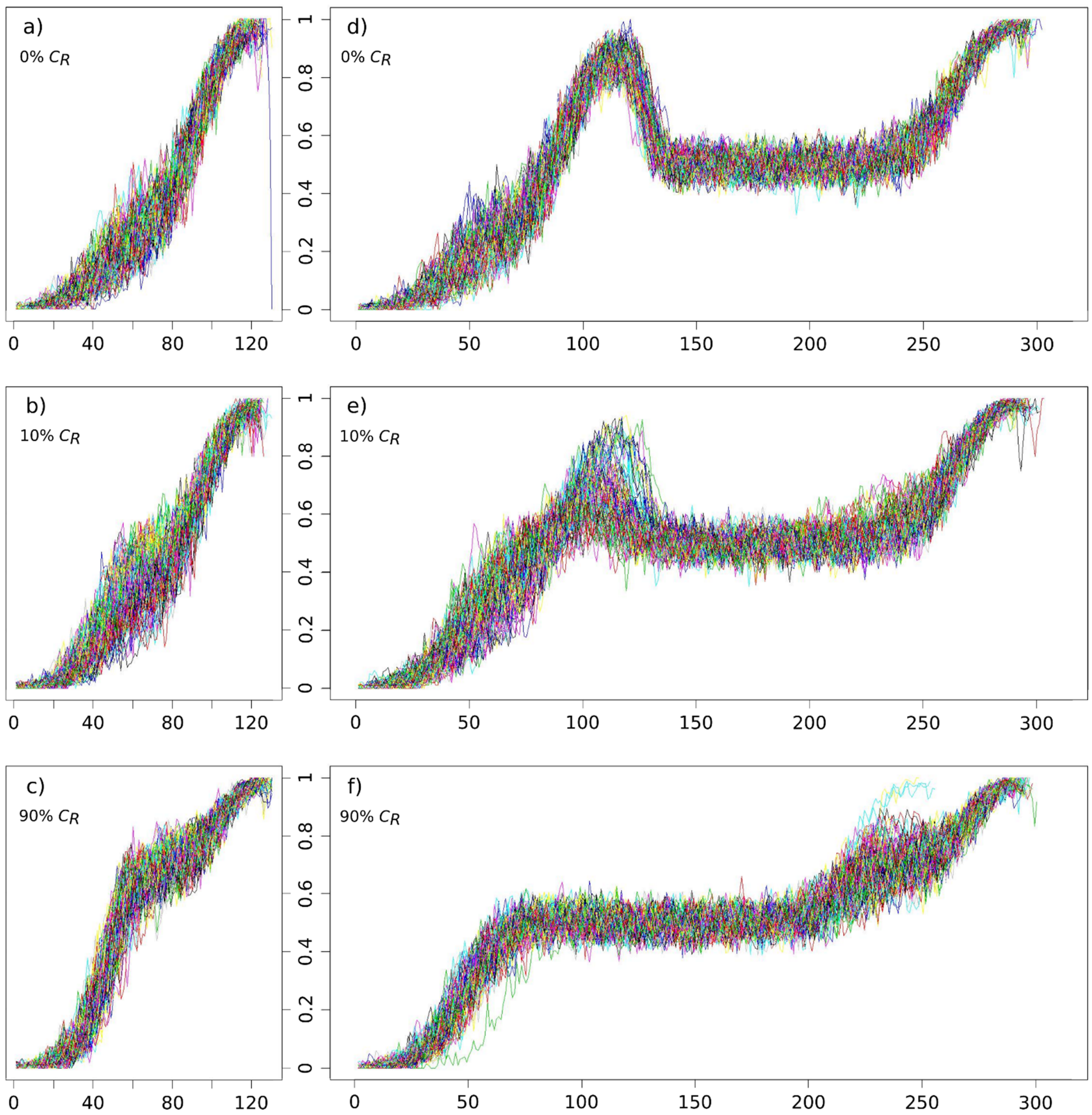

Fig S5. Masculinization rate. X axis: years of climate warming. Y axis: relative frequency of phenotypic males among genetically female individuals. On the left: scenarios 0% C_R (a), 10% C_R (b) and 90% C_R (c) starting with XX/XY system (female genotypes: *Aa, aa*). On the right: scenarios 0% C_R (d), 10% C_R (e) and 90% C_R (f) starting with ZW/ZZ system (female genotype: *aa*). Curves indicate separate runns (100 repeated runs per scenario).

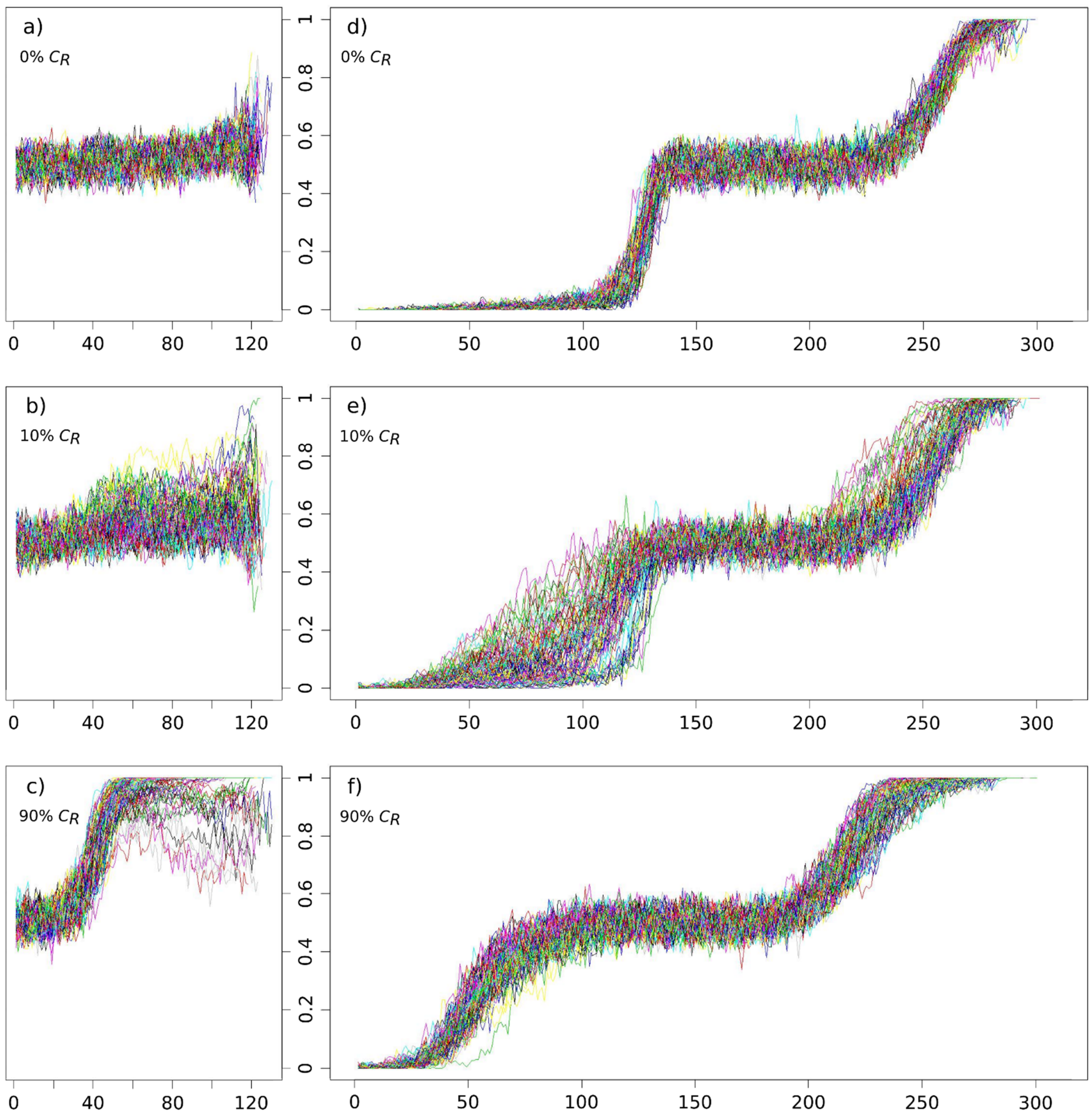

Fig S6. Relative frequency of genotype *aa* among adults. X axis: years of climate warming. Y axis: relative frequency. On the left: scenarios 0% C_R (a), 10% C_R (b) and 90% C_R (c) starting with XX/XY system. On the right: scenarios 0% C_R (d), 10% C_R (e) and 90% C_R (f) starting with ZW/ZZ system. Curves indicate separate runs (100 repeated runs per scenario).

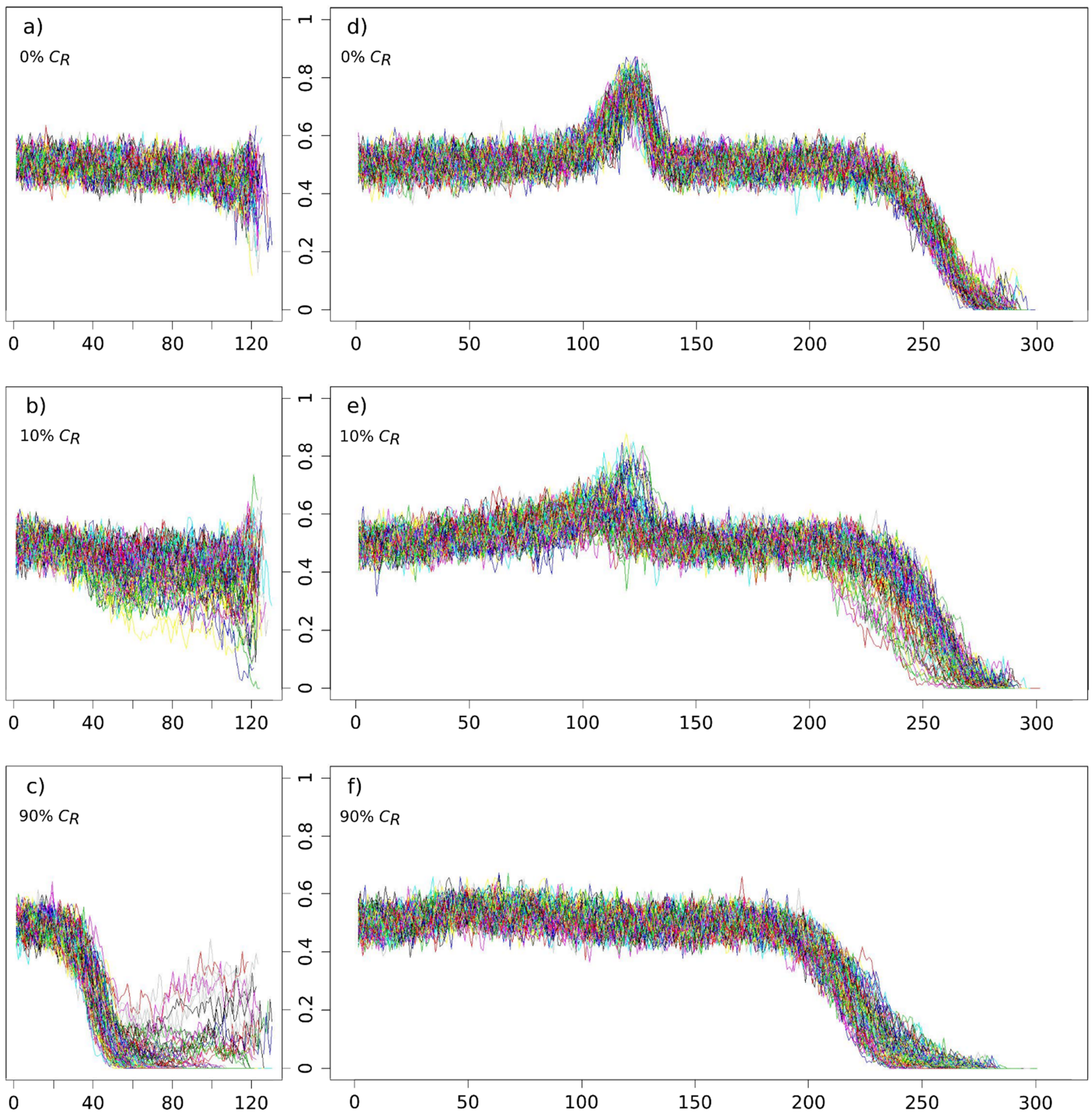

Fig S7. Relative frequency of genotype *Aa* among adults. X axis: years of climate warming. Y axis: relative frequency. On the left: scenarios 0% C_R (a), 10% C_R (b) and 90% C_R (c) starting with XX/XY system. On the right: scenarios 0% C_R (d), 10% C_R (e) and 90% C_R (f) starting with ZW/ZZ system. Curves indicate separate runs (100 repeated runs per scenario).

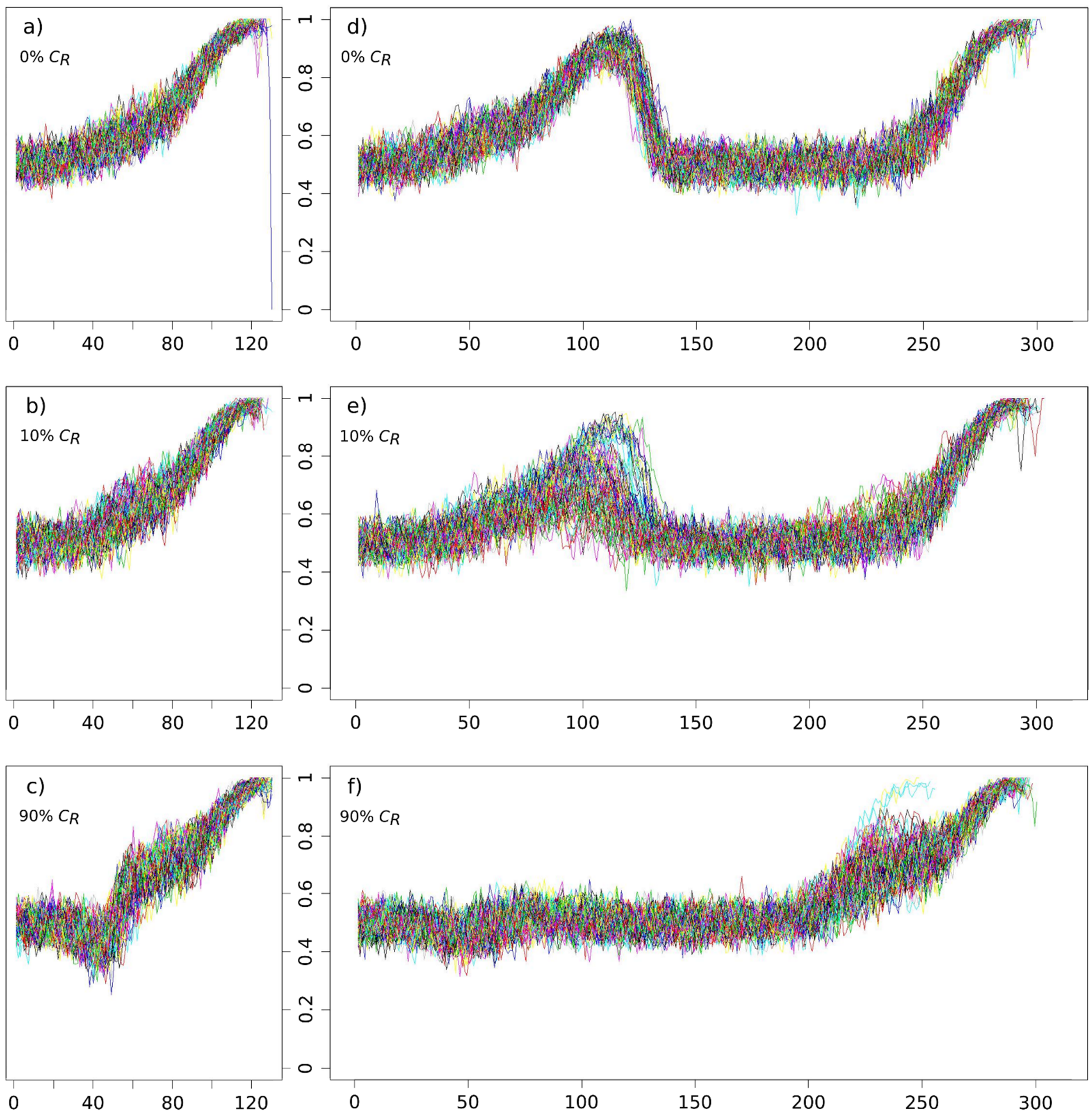

Fig S8. Adult sex ratio. X axis: years of climate warming. Y axis: proportion of males among adults. On the left: scenarios 0% C_R (a), 10% C_R (b) and 90% C_R (c) starting with XX/XY system. On the right: scenarios 0% C_R (d), 10% C_R (e) and 90% C_R (f) starting with ZW/ZZ system. Curves indicate separate runs (100 repeated runs per scenario).

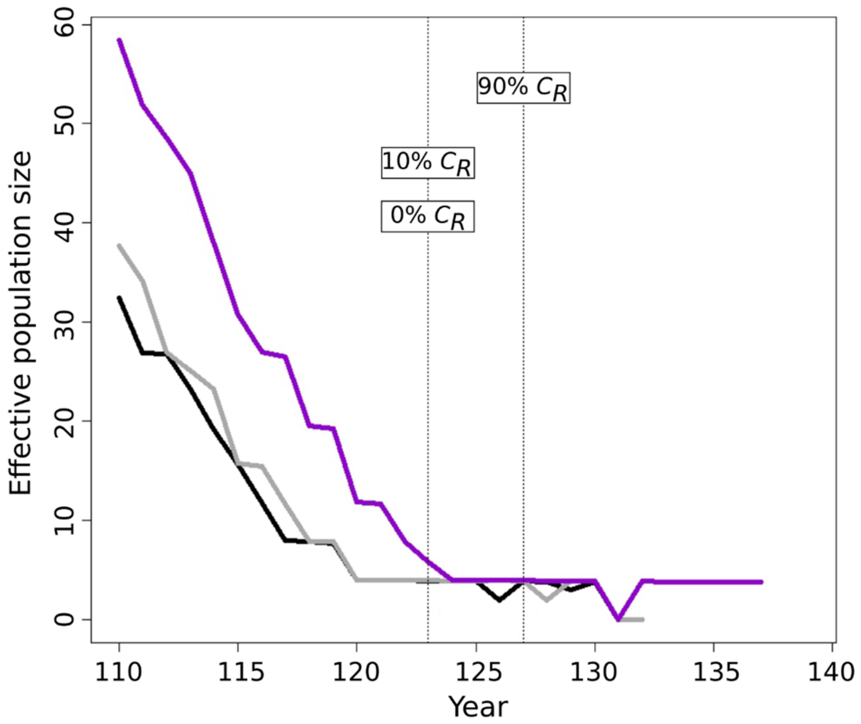

Fig S9. Median effective population size during the last decades before extinction in originally XX/XY populations. Years denote the number of years since the start of climate warming. Median effective population size calculated from the persisting populations out of a total of 100 in each year are shown for each scenario: 0% C_R (black), 10% C_R (grey) and 90% C_R (prurple). Vertical dotted lines indicate the median extinction time for each scenario (note: this is the same in scenarios 0% C_R and 10% C_R).
