# Supplementary material for: Evolutionary and demographic consequences of temperature-induced masculinization under climate warming: the effects of mate choice
Source: BMC Ecol Evol. 2021 Feb 4;21:16. doi: 10.1186/s12862-021-01747-3 (PMC7860201; doi:10.1186/s12862-021-01747-3)

Supporting Information 5 – Sensitivity analysis

Evolutionary and demographic consequences of temperature-induced masculinization under climate warming: the effects of mate choice

Nemesházi E[1,2,3], Kövér Sz[2], Bókony V[1]

[1] Lendület Evolutionary Ecology Research Group, Plant Protection Institute, Centre for Agricultural Research, Eötvös Loránd Research Network, Herman Ottó út 15, 1022 Budapest, Hungary. E-mail: nemeshazi.edina@atk.hu, bokony.veronika@atk.hu

[2] Conservation Genetics Research Group, Department of Ecology, University of Veterinary Medicine Budapest, István utca 2, 1078 Budapest, Hungary

[3] Konrad Lorenz Institute of Ethology, Department of Interdisciplinary Life Sciences, University of Veterinary Medicine, Savoyenstr. 1a, A-1160 Vienna, Austria

Table of contents:

Figure S10. Intermediate inheritance with two strong preference alleles (C_R=0.1, C_N=0.9)

Figure S11. C_R versus no preference (C_R=0.1, C_N=0.5; dominant/recessive)

Figure S12. C_R versus no preference (C_R=0.1, C_N=0.5), intermediate inheritance

Figure S13. Multiallelic threshold

Figure S14. 75% WW survival

Figure S15. 50% WW survival

Figure S16. 25% WW survival

Figure S17. WW lethality

General information:

Figures in this document show the results of simulations that we ran to assess sensitivity of our results described in the main text for certain parameter settings. All figures show median values calculated from 100 runs. Vertical dotted lines indicate the end of each sex-determination period:

- in simulations starting with XX/XY system: XX/XY, and final period
- in simulations starting with ZW/ZZ system: ZW/ZZ, transition, XX/XY, and final period

Dashed lines indicate the start of ultimate population decline.
For comparison with the main results, see Figure 1 (ASR, relative frequencies of genotypes and threshold alleles) and Figure 5 (relative frequency of C_R allele) in the main text.

Figure S10. Intermediate inheritance with two strong preference alleles (C_R=0.1, C_N=0.9)

In this situation, the expression of female preference is distributed in the starting populations as follows:

- 10% C_R scenario: 1% prefer sex-reversed males, 18% show no preference, 81% prefer normal males
- 90% C_R scenario: 81% prefer sex-reversed males, 18% show no preference, 1% prefer normal males

The results (ASR, relative frequencies of sex-chromosome genotypes and threshold alleles, timing of transitions and extinction) are very similar to the results shown in the main text (dominant/recessive inheritance), except for one difference: starting with a rare C_R in an XX/XY system, C_R does not disappear (although its frequency decreases steadily).

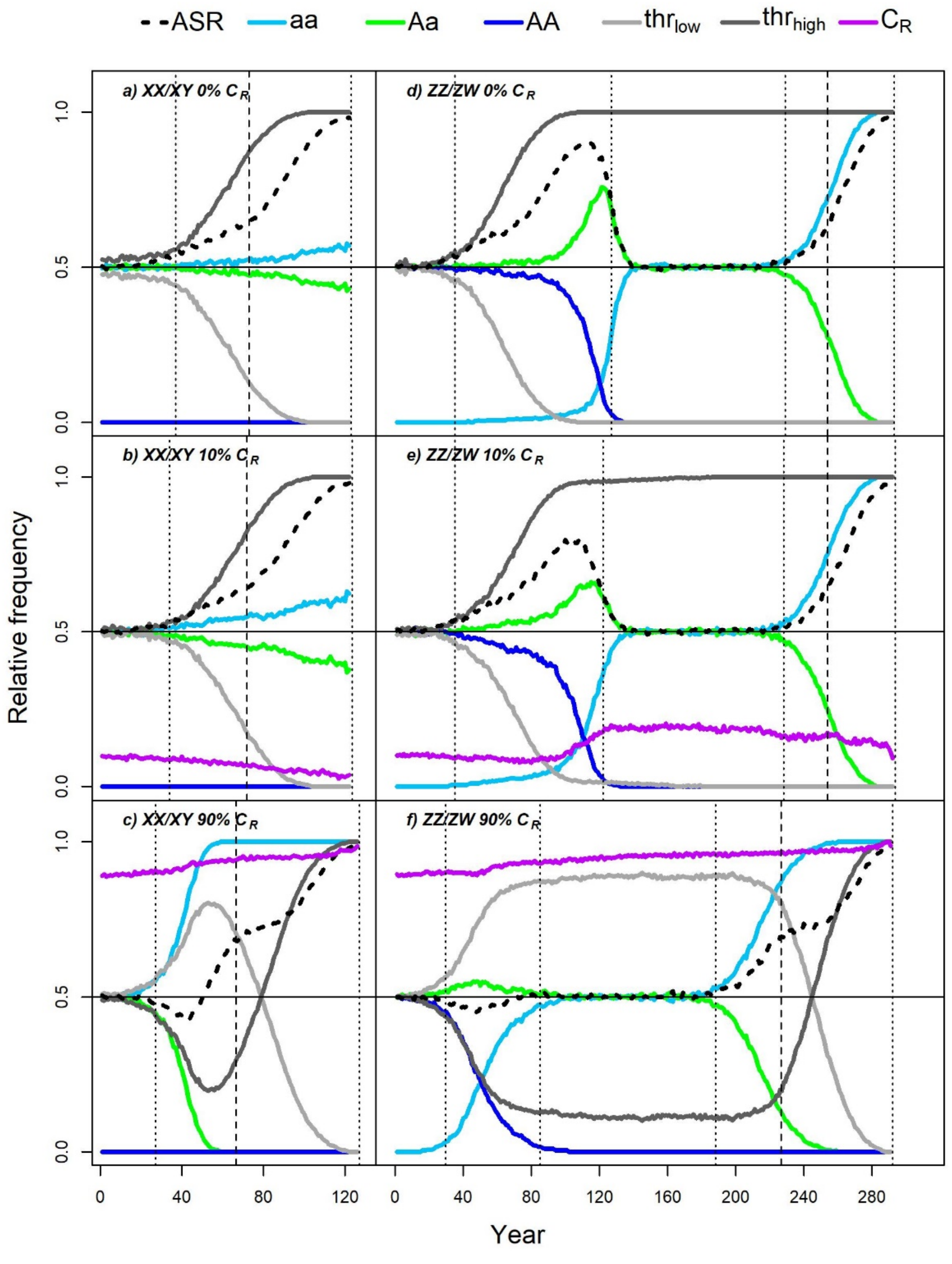

Figure S11. C_R versus no preference (C_R=0.1, C_N=0.5; dominant/recessive)

In this situation, the expression of female preference is distributed in the starting populations as follows:

- 10% C_R scenario: 19% prefer sex-reversed males, 81% show no preference
- 90% C_R scenario: 81% prefer sex-reversed males, 19% show no preference

In these scenarios, the lack of preference for normal males results in faster extinction of the original male genotype, later spread of thr_{high}, and less marked ASR bias towards males during the ZZ/ZW-XX/XY transition compared to the scenarios in the main text. Also, the frequency of C_R shows little change over time in the 10% C_R scenarios (it is not selected against when rare because the majority of the females are indiscriminate). However, the effects of C_R on ASR and transition times are very similar to the scenarios in the main text.

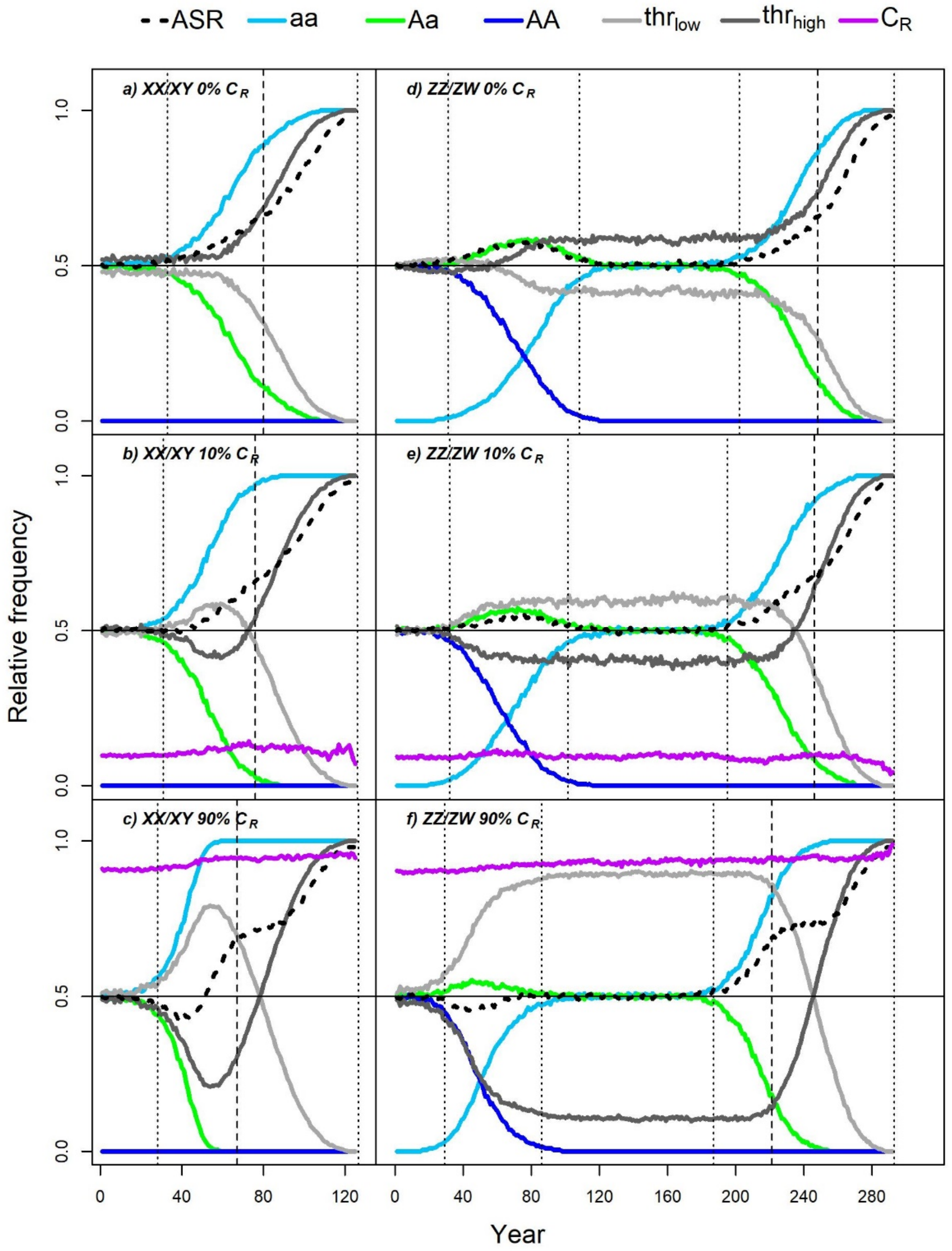

Figure S12. C_R versus no preference (C_R=0.1, C_N=0.5), intermediate inheritance

In this situation, the expression of female preference is distributed in the starting populations as follows:

- 10% C_R scenario: 1% prefer sex-reversed males with 0.9 probability, 18% prefer sex-reversed males with 0.7 probability, 81% show no preference
- 90% C_R scenario: 81% prefer sex-reversed males with 0.9 probability, 18% prefer sex-reversed males with 0.7 probability, 1% show no preference

These results are very similar to the previous case (C_R versus no preference; dominant/recessive). Thus, the frequency of C_R changes very slightly or not at all when it is "competing" with a no-preference allele; this result does not depend on the type of inheritance. However, the effects of C_R on ASR and transition times are very similar to the scenarios in the main text.

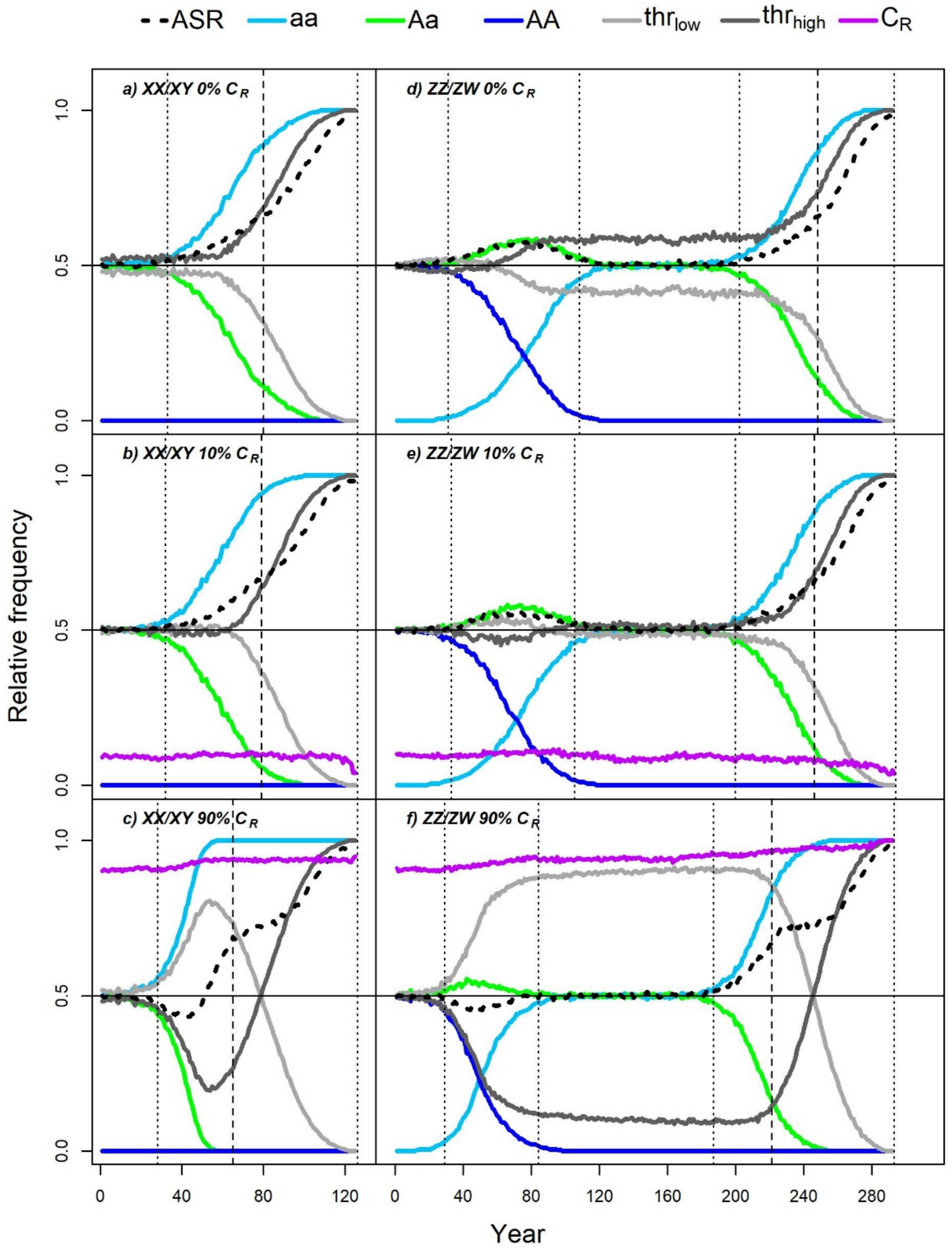

Figure S13. Multiallelic threshold

The number of *thr* alleles was set to 10, such that each allele started with a frequency of 0.1, and their values were evenly distributed between the lowest and highest value used in the main-text simulations.

The results are similar to those in the main text. Note: in the graphs below, solid grey curves show the frequency of the lowest and highest *thr* allele (from the original 10 alleles).

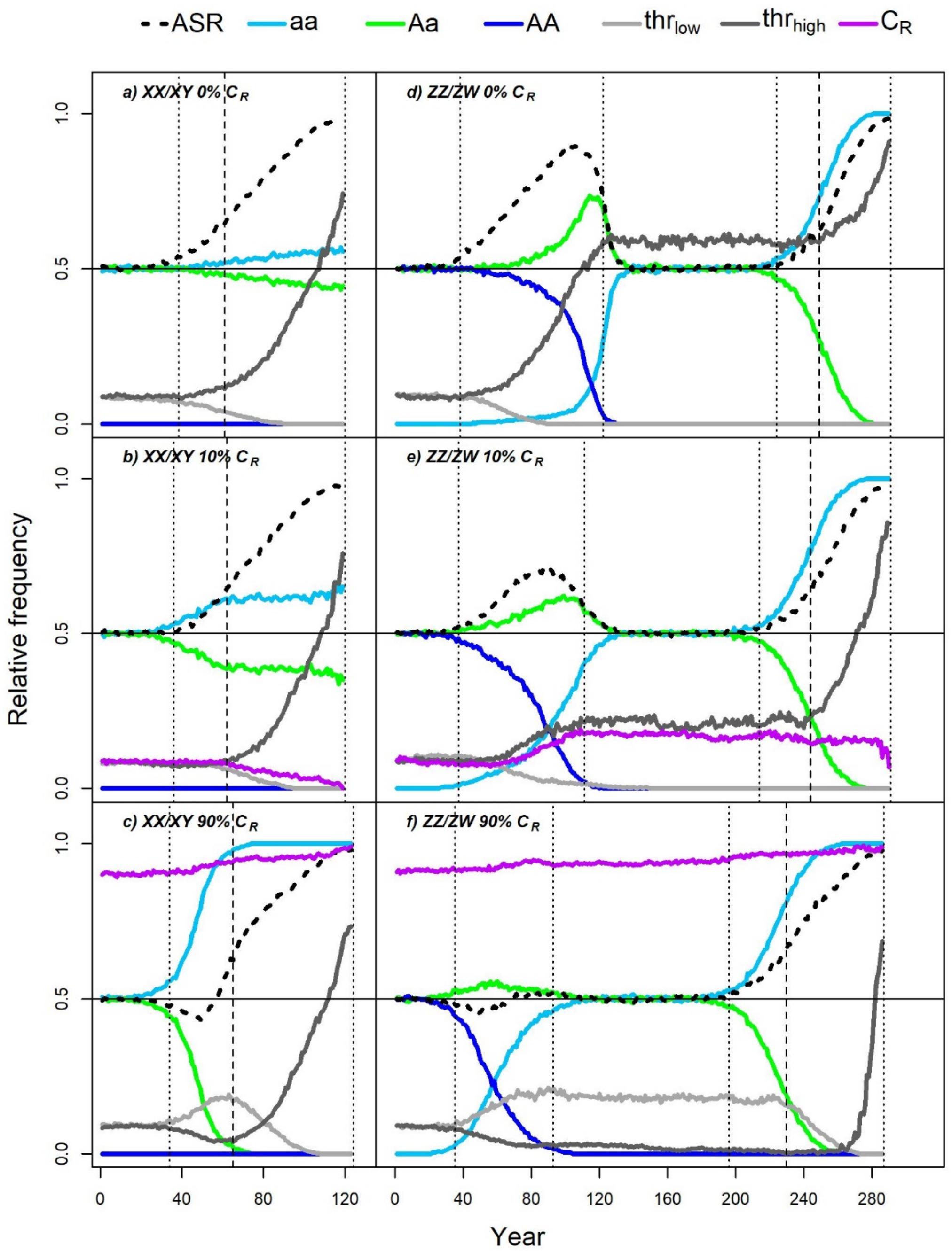

Scenarios with reduced WW viability

Figure S14. 75% WW survival

When the survival of WW offspring is 75% of the survival of other genotypes, the spread of C_R and its effects are similar to the scenarios with 100% WW viability: C_R reduces the ASR bias and speeds up the transition. Because of the higher mortality of WW individuals (which are more likely to develop into phenotypic females), the newly evolved XX/XY system maintains a somewhat male-biased ASR until system destabilization.

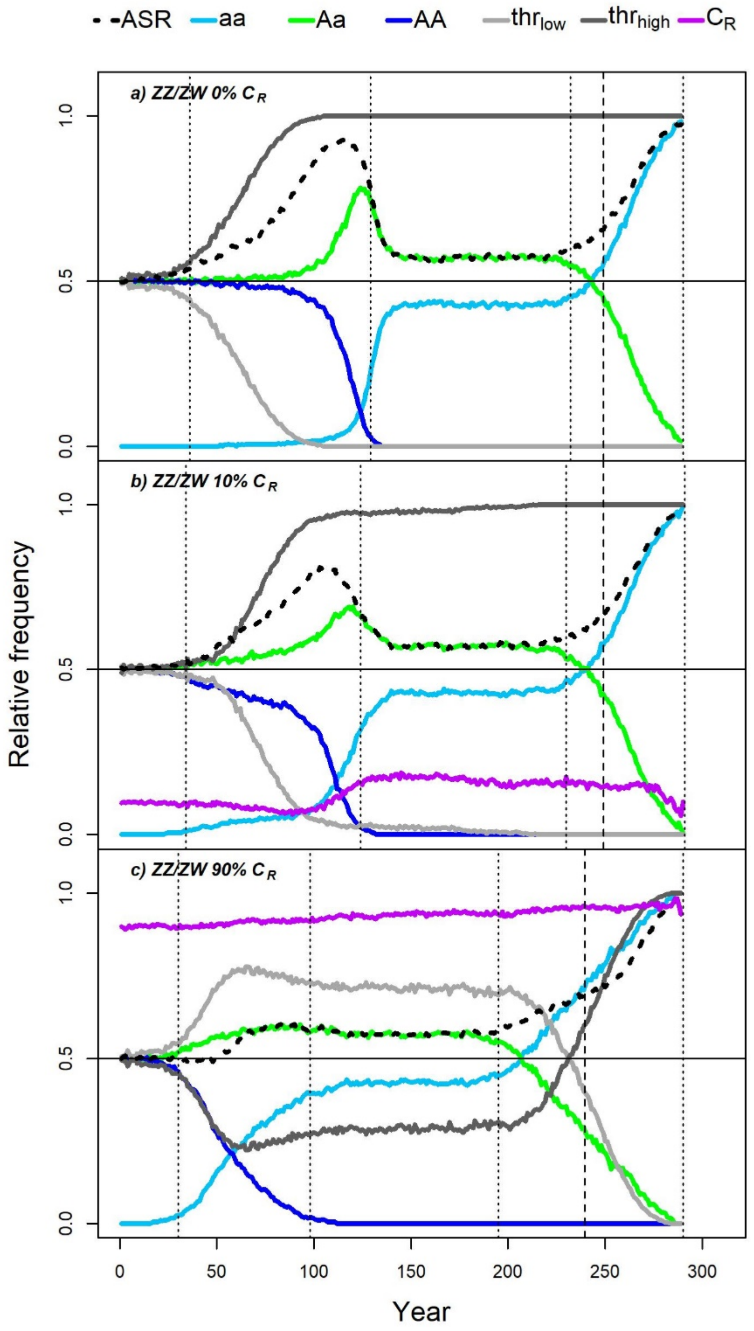

Figure S15. 50% WW survival

Again, the effects of C_R are similar to the 100% WW viability scenarios. Relative frequency of the widespread C_R allele increases only slightly, whereas the rare C_R allele disappears, although it increases for a short time when ASR becomes highly male-biased before transition. After transition, ASR remains relatively male-biased.

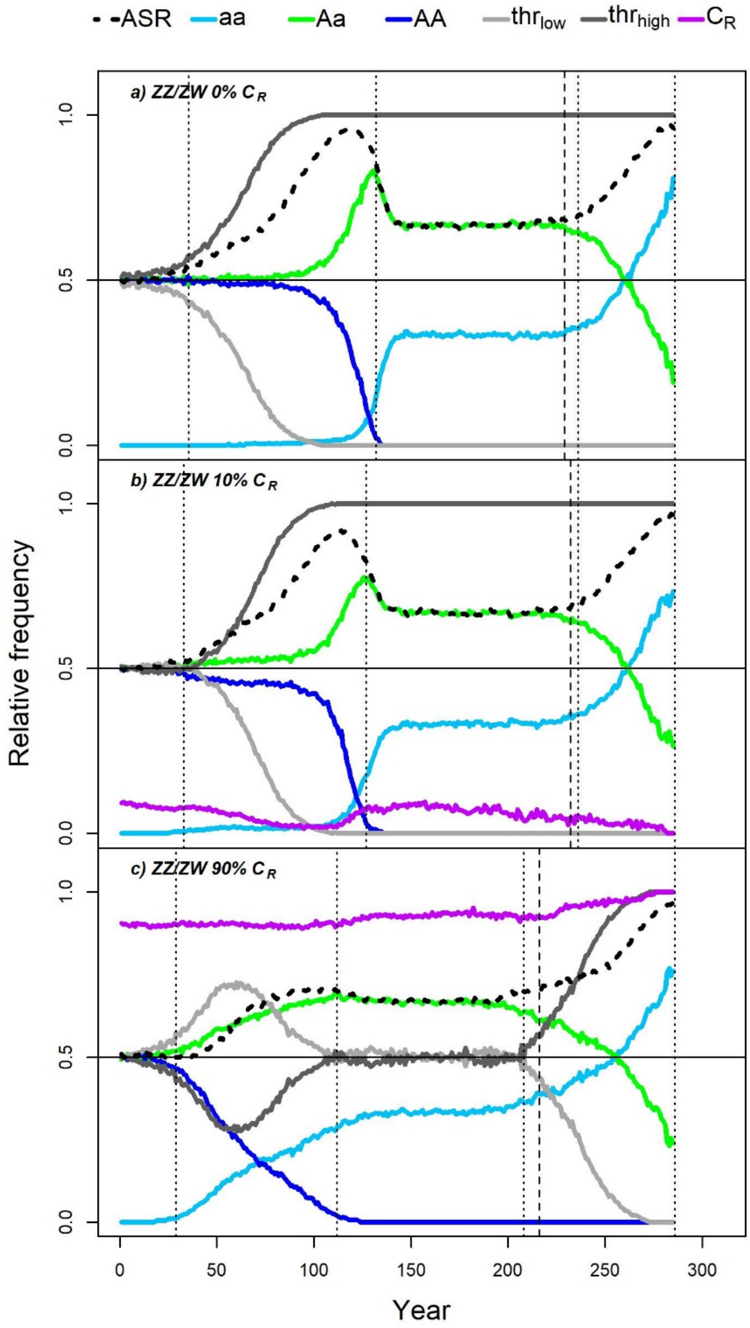

Figure S16. 25% WW survival

When the survival of WW offspring is only 25% of the survival of other genotypes, most of the populations cannot transition to an XX/XY system and die out after ca. 120 years when no C_R allele is present. However, the presence of C_R saves the population from this early extinction by enabling the switch to XX/XY system (in slightly more than half of the runs when C_R is rare; always when C_R is widespread; see Fig. 5 in the main text). This switch happens rapidly when ASR has become highly male-biased; during this short time the relative frequency of C_R increases. After transition, the population maintains a strongly male-biased ASR.

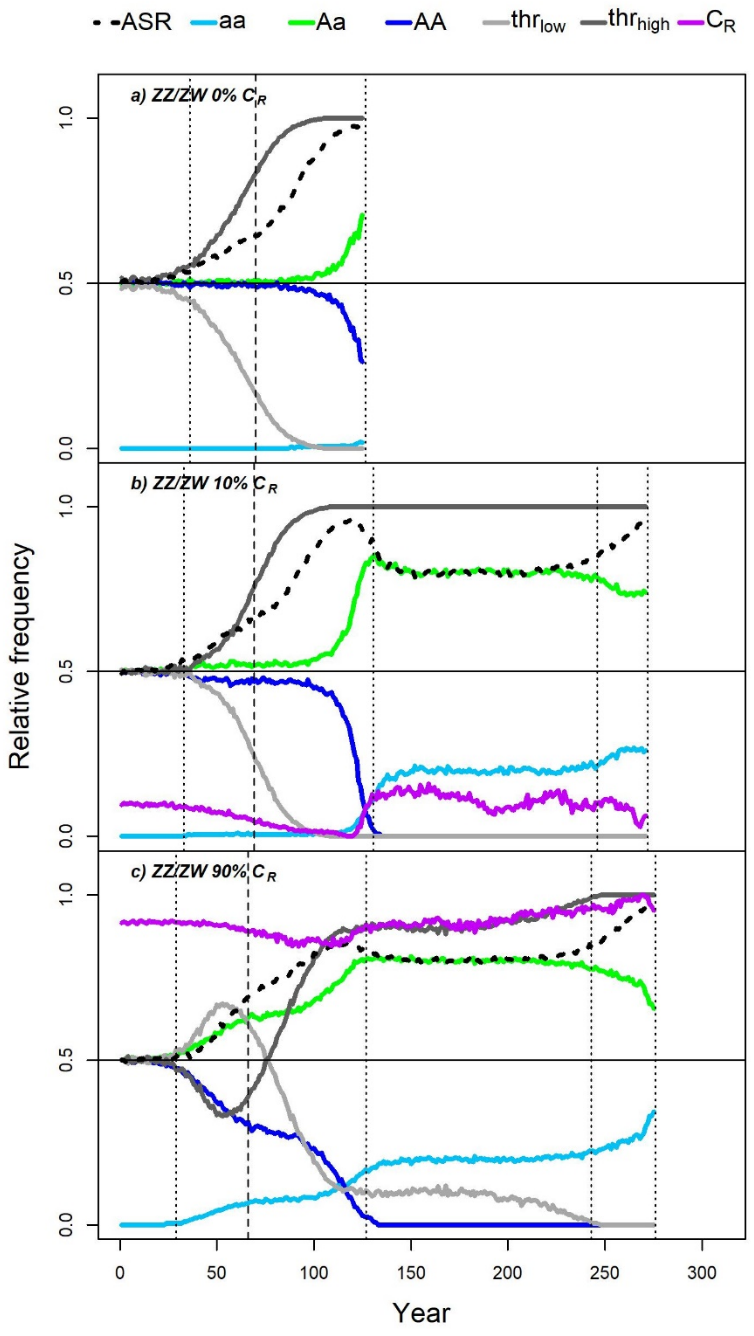

Figure S17. WW lethality

WW lethality makes the ZZ/ZW system behave similarly to the XX/XY system: no transition from ZZ/ZW to XX/XY, and the population dies out after ca. 120 years. The effects of C_R are also similar: it has little effect on ASR change or extinction time, but it makes the starting system destabilize slightly earlier than when no C_R is present. The only difference is that C_R never spreads.

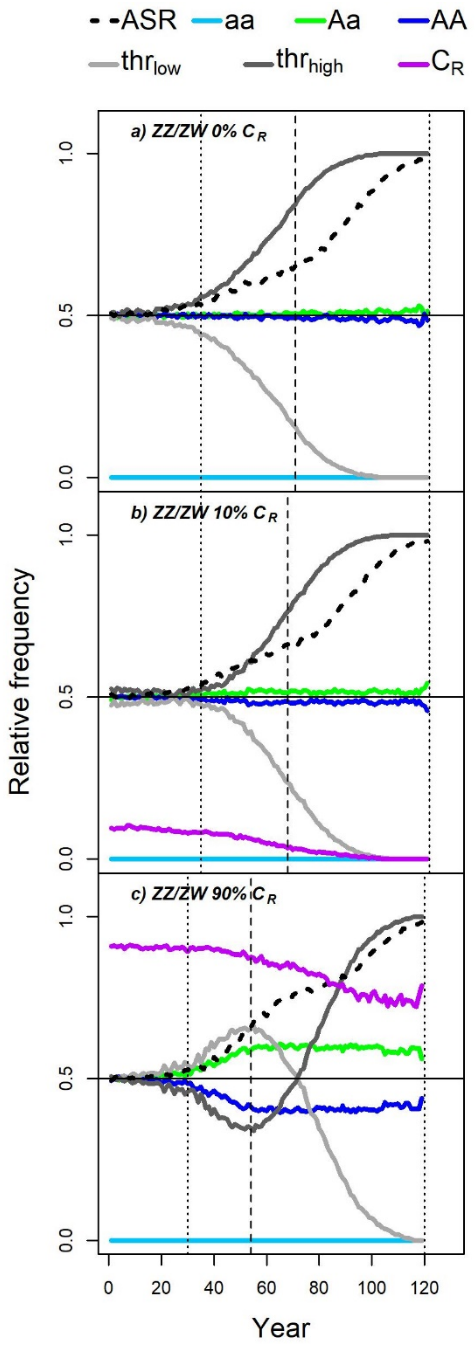

Supplement: Supplementary file 5 — Additional file 5. Sensitivity analysis. [file 12862_2021_1747_MOESM5_ESM.pdf]
